# Altered resting-state functional connectivity in hiPSCs-derived neuronal networks from schizophrenia patients

**DOI:** 10.3389/fcell.2022.935360

**Published:** 2022-09-07

**Authors:** Sofía Puvogel, Kris Blanchard, Bárbara S. Casas, Robyn L. Miller, Delia Garrido-Jara, Sebastián Arizabalos, Stevens K. Rehen, Magdalena Sanhueza, Verónica Palma

**Affiliations:** ^1^ Laboratory of Stem Cells and Developmental Biology, Department of Biology, Faculty of Sciences. Universidad de Chile. Santiago, Chile; ^2^ Cell Physiology Laboratory, Department of Biology, Faculty of Sciences, Universidad de Chile, Santiago, Chile; ^3^ Department of Computer Science, Georgia State University, Atlanta, GA, United States; ^4^ Tri-Institutional Center for Translational Research in Neuroimaging and Data Science (TReNDS Center), Atlanta, GA, United States; ^5^ Instituto D’Or de Pesquisa e Ensino (IDOR), Rio de Janeiro, Brazil

**Keywords:** schizophrenia, neural stem cells (NSCs), hiPSCs, resting-state functional connectivity, calcium imaging, neurodevelopment

## Abstract

Schizophrenia (SZ) is a severe mental disorder that arises from abnormal neurodevelopment, caused by genetic and environmental factors. SZ often involves distortions in reality perception and it is widely associated with alterations in brain connectivity. In the present work, we used Human Induced Pluripotent Stem Cells (hiPSCs)-derived neuronal cultures to study neural communicational dynamics during early development in SZ. We conducted gene and protein expression profiling, calcium imaging recordings, and applied a mathematical model to quantify the dynamism of functional connectivity (FC) in hiPSCs-derived neuronal networks. Along the neurodifferentiation process, SZ networks displayed altered gene expression of the glutamate receptor-related proteins *HOMER1* and *GRIN1* compared to healthy control (HC) networks, suggesting a possible tendency to develop hyperexcitability. Resting-state FC in neuronal networks derived from HC and SZ patients emerged as a dynamic phenomenon exhibiting connectivity configurations reoccurring in time (hub states). Compared to HC, SZ networks were less thorough in exploring different FC configurations, changed configurations less often, presented a reduced repertoire of hub states and spent longer uninterrupted time intervals in this less diverse universe of hubs. Our results suggest that alterations in the communicational dynamics of SZ emerging neuronal networks might contribute to the previously described brain FC anomalies in SZ patients, by compromising the ability of their neuronal networks for rapid and efficient reorganization through different activity patterns.

## Introduction

Schizophrenia (SZ) is a chronic mental disorder that affects over 20 million people worldwide (World Health Organization, 2019). The neurodevelopmental hypothesis of SZ proposes that an abnormal developmental trajectory converges into vulnerable brain circuits, leading the organism prone to an increased likelihood of developing psychosis when faced with stressful events during adulthood (Fatemi and Folsom, 2009). Nonetheless, the mechanisms triggering and predicting the evolution of the disease remain poorly understood.

Different studies associate SZ symptomatology with an altered communication across different brain regions [reviewed in (Fitzsimmons et al., 2013)]. In this context, functional connectivity (FC) analysis, which calculates the temporal correlation between spatially remote events of brain activity, has been widely used to evaluate communication across the brain. One of the most common techniques used to measure regional brain activity is functional magnetic resonance imaging (fMRI) (Schölvinck et al., 2010), which registers the hemodynamic variation in response to changes in brain activity. This methodology allows quantifying the temporal correlation between signals originating from different parcellated brain nodes (voxels); a measure that reflects how strongly different brain regions communicate. fMRI studies have shown abnormal brain FC in SZ patients in both resting-state (Garrity et al., 2007; Whitfield-Gabrieli et al., 2009; Sheffield and Barch, 2016; Erdeniz et al., 2017) and during task performance (Whitfield-Gabrieli et al., 2009; Sheffield and Barch, 2016; Godwin et al., 2017). Some of the resting-state studies assumed that FC remains static during the data acquisition time period (∼5–15 min) (Menon and Krishnamurthy, 2019); however, it has been demonstrated that brain resting-state FC is a non-stationary phenomenon. Instead, it characterizes by exploring different connectivity states (Cabral et al., 2017; Geng et al., 2020). Furthermore, the flow-through of these configurations is not an arbitrary process, since discrete connectivity states consistently recur along with the scans (Hutchison et al., 2013). These findings promoted high interest in understanding the dynamics of FC in health and disease, and thus, in the development of novel analytic approaches to study the variations in FC in the resting brain [discussed in (Cabral et al., 2017)]. Analysis of the resting-state FC course in patients with SZ depicted a pronounced reduction in the number and diversity of connectivity states when compared to healthy individuals, reflecting alterations in brain communicational dynamics associated with SZ (Miller et al., 2016).

FC measurements in SZ are drawn primarily from fMRI scanning data obtained from adults diagnosed with the disease. Since fMRI relies on hemodynamic fluctuations related to neural activity, it is not suitable for measuring brain activity with high temporal resolution; thereby, it may miss fast transitions in brain FC configurations. Additionally, the spatial resolution of this technique is low. The typical size of fMRI voxels is around 3 × 3 × 3 mm^3^, reflecting the average activity of hundreds to thousands of neurons (Arthurs and Boniface, 2002). Such volume covers the entire cortical thickness, containing several cell types with distinct morphological and functional properties. Thus, there is still a gap in our understanding of the cellular mechanisms involved in brain FC anomalies in SZ.

Synapses are the elementary structural and functional units supporting information flow between individual neurons across the brain. Accordingly, the study of neuronal communication at the cellular level may increase our understanding of FC alterations observed in patients with SZ. Human-induced pluripotent stem cells (hiPSCs)-derived models reflect the entire genetic component of patients with complex polygenic brain disorders, (Vadodaria et al., 2020) such as SZ. These cells can faithfully recapitulate neurogenesis and the evolution of spontaneous network activity during brain development (Mariani et al., 2012; Brennand et al., 2014; Kirwan et al., 2015; Paşca et al., 2015; Izsak et al., 2019; Fair et al., 2020); therefore, these cultures represent a novel strategy to obtain patient-derived neurons and study the underlying neuronal mechanisms of developmental psychiatric diseases. While so far it is not possible to reproduce the complexity of specific brain regions and their connectivity patterns, this *in-vitro* neuronal model could facilitate the exploration of general properties of FC dynamics in emerging neuronal networks. With this aim, we differentiated hiPSCs derived from patients with SZ and healthy control donors (HC) into long-term neuronal cultures. Using Ca^2+^ imaging, we visualized local changes in fluorescence associated with spontaneous activity in large neuronal populations and measured the temporal correlation between signals arising from individual neurons. We adapted a FC analysis methodology, previously designed to study time-varying discrete connectivity states at whole brain level in SZ (Miller et al., 2016), to quantify resting-state FC dynamics at the cellular level. As compared to the networks derived from HC, neuronal networks derived from SZ patients exhibited both a reduced ability to explore different functional connectivity configurations and a reduced competence for rapid connectivity reshaping, suggesting that alterations in neuronal communicational dynamics are already present during early development in SZ and may contribute to the brain FC anomalies described in SZ patients. These observations could have global implications for understanding the communicational properties of the brain in patients with SZ and may open new routes for exploring fundamental principles that rule brain communication through different spatiotemporal scales, in normal and pathological contexts.

## Methods and materials

### Description of the donors and cell lines

The hiPSCs lines used in this study were obtained from three healthy controls and four SZ donors, all of whom displayed a normal karyotype. Detailed information about donors and samples is presented in Supplementary Table S1. All the SZ patients presented a high probability of a genetic predisposition to SZ, based on the existence of relatives with the disease. Additionally, to decrease genetic heterogeneity among the cell lines, two of the donors are siblings. SZ#1 is a male with paranoid schizophrenia, while his sister SZ#2 was diagnosed with schizoaffective disorder and presented a history of drug abuse (cell lines are available at Coriell (Brennand et al., 2011)]. Donors SZ#3 and SZ#4 are males who were diagnosed with SZ according to the DSM-IV Axis IV criteria, both exhibiting resistance to clozapine, with a family history of SZ and no major psychiatric comorbidities, no history of head trauma and no drug or alcohol abuse. The cell lines from SZ#3 and SZ#4 were reprogrammed at D’Or Institute for Research and Education (Sochacki et al., 2016). Considering the high likelihood of a genetic risk component to the disease within the patients’ family members, the selected controls are not related to the patients. HC#1 is a female and the cell line is available at Coriell. HC#2 and HC#3 are both males and the cell lines were reprogrammed at the D’Or Institute for Research and Education. The establishment of hiPSCs and derivation of Neural Stem Cells (NSCs) was carried out according to the international standards and the approval of the Research Ethics Council “Comité de Ética em Pesquisa–Hospital Copa D’Or” [Certificado de Apresentação de Apreciação Ética (CAAE): 32385314.9.0000.5249].

### Differentiation of NSCs to mature neuronal cultures

hiPSCs-derived NSCs were kindly donated by Dr. Stevens Rehen from the D’Or Institute and Federal University of Rio de Janeiro. Differentiation of hiPSCs to NSCs was performed as described in (Casas et al., 2018). NSCs differentiation to mature neuronal networks was conducted by adapting the protocol of Shi et al. (2012). NSCs were thawed and seeded in Geltrex-coated plastic 60 mm plates and maintained in Neural Expansion Media (NEM; DMEM/F12 and Neurobasal medium (1:1) plus Neural Induction Supplement; Thermo Fisher Scientific, Carlsbad, CA, United States) until they reached 80%–100% confluence. Next, NSCs were detached from the plate with accutase (7 min/37°C), centrifugated at 300 *g* for 4 min and resuspended in NEM plus 10 µM Rock Inhibitor (Y-27632; Merck, Darmstadt, Germany). 1*10^6 cells were plated in 35 mm petri dishes coated by poly-l-ornithine/laminin (10 μg/ml and 2.5 μg/ml, respectively). After 48 h, the medium was replaced by Neural Differentiation Media (NDM; DMEM/F12 and Neurobasal medium (1:1) supplemented with 1 × N2, 1 × B27 and 2-Mercaptoethanol 100 μM). NDM was changed every 2 days. 14–16 days after plating, cells were detached with accutase and passed in a 1:3 ratio into 35 mm poly-l-ornithine/laminin-coated plates. Medium changes were done every 2 days. Around day 30, cells were passed into their final coated 35 mm plate, in a 1:4 ratio (∼500.000 cells per 35 mm petri dish). Cells were maintained in NDM, with medium changes every other day, for 60 more days and were supplemented with 10 µM Rock inhibitor for 48 h, after every passage.

### Immunofluorescence

At 60 or 100 days of differentiation, cells were fixed with paraformaldehyde 4%, permeated with Triton-X (Sigma, St. Louis, MO, United States) 0.2%, blocked with BSA 5% in PBST (tween-20 0.1% in PBS), and incubated overnight at 4°C with anti-tau1 (mouse; #MAB3420 Milipore), anti-synaptophysin (mouse; #101011 Synaptic Systems), anti-MAP2 (rabbit; #AB5622 Millipore), anti-homer-1 (rabbit; #160002 Synaptic Systems), anti-GFAP (rabbit; #G9269 Sigma), and anti-TUJ1 (mouse; Sigma T8660). Subsequently, cells were incubated with secondary antibodies (goat anti-mouse Alexa 488, goat anti-rabbit Alexa 555, Invitrogen). Nuclei were stained with 4′-6-diamino-2-phenylindole (DAPI) 1 μg/ml for 5 min. Images were acquired with a Zeiss LSM 710 confocal microscope.

### Electrophysiology

Whole-cell voltage-clamp recordings were performed at 60 days of differentiation in HC and SZ derived cultures. Neurons were selected following morphological criteria. Na^+^ inward and K^+^ outward currents were evoked by voltage steps ranging from −100 to +40 mV in 10 mV increments (Vhold = −75 mV). Data were acquired at 50 kHz and low-pass filtered at 2.9 kHz, by an EPC-10 amplifier (HEKA Elektronik GmbH, Reutlingen, Germany). Patch electrodes (∼4.5 MΩ) were pulled from borosilicate. The internal solution contained (in mM): 135 K-gluconate, 2 MgCl_2_, 2 Na_2_ATP, 0.3 NaGTP, 10 HEPES, 7 NaCl (pH 7.4). The recording chamber was continuously perfused at 1–2 ml/min with ACSF solution containing (in mM): 115 NaCl, 2.5 KCl, 1.3 NaH_2_PO_4_, 26 NaHCO_3_, 25 glucose, 5 Na-Pyruvate, 2 CaCl_2_ and 1 MgCl_2_ (300 mOsm/kg), gassed with 5% CO_2_/95% O_2_ (pH 7.4). Recordings were performed at room temperature (20°C–25°C). Series resistance (10 MΩ–40 MΩ) were not compensated and recordings were discarded for variations higher than 20% along the experiment.

### qPCR

For qPCR analysis, we used neuronal cultures derived from three SZ (#1,3,4) and three HC (#1–3) cell lines. Cellular cultures from early differentiated cells (30 days) and late differentiated cells (range of 70–91 days, herein defined as 90 days) were stored in Trizol (Thermo Fisher Scientific, Carlsbad, CA, United States) at −80°C. Samples obtained from the same cell line but from independent differentiation procedures were pooled together to extract total RNA. cDNA was synthesized from 1 μg RNA, using an M-MLV reverse-transcription kit (Promega, Madison, WI, United States). Primers were specifically designed to measure the expression of the genes listed in Supplementary Figure S1A. Relative gene expression was assessed by qPCR (Agilent Technologies Thermocycler, Santa Clara, CA, United States). mRNA levels were calculated as the fold-change expression via 2^−ΔΔCt^ and gene expression was normalized to three different housekeeping genes (*B2M, 18S, GAPDH*)*.* Fold changes were assessed relative to a control sample (HC #1) or differentiation time.

### Conditioned medium (CM) collection and neuro-proteomic profile

The presence of 30 different neuronal growth factors in serum-free collected CM (48 h) from cell cultures of 75 days of differentiation [four SZ (#1–4) and three HC (#1–3)], was evaluated using the Human Neuro Antibody Array II (#ab211063, Abcam, Cambridge, United Kingdom). Spots were detected by chemo-luminescence and intensity was quantified by densitometry (ImageJ, NIH, United States). The levels of each factor were measured in duplicates and normalized to internal controls provided by the assay.

### Statistical analysis for mRNA expression and neuro-proteomic profile comparisons

mRNA fold-change normality was assessed with D’Agostino-Pearson and group comparison was done using a Nested t-test and linear mixed effect modelling, with a random intercept for cell line identity (id) (Supplementary Table S2). Group comparison in terms of the secreted proteins, measured with the neuro-proteome array, was also performed with linear mixed effect modelling. To evaluate the significance of the regression coefficients associated with the diagnosis (group), we used a Z test and Bonferroni correction for multiple comparisons. Statistical significance was set at *p* < 0.05.

### Calcium imaging

Ca^2+^ transients were recorded from single cells across different regions of the plate showing high cellular density at 80–90 days of differentiation, using the cell-permeant Ca^2+^ indicator Oregon Green™ 488 BAPTA-1 (OGB-1 AM; peak absorption = 493 nm; Thermo Fisher Scientific, Carlsbad, CA, United States). Epifluorescence imaging was performed with a mercury arc lamp and using a band pass excitation (450–490 nm) filter. Emitted light was detected with an electron-multiplying CCD camera (High Performance CCD Sensicam, PCO Cooke) with a band pass filter (515–565). Three SZ (#2, 3, and 4) and two HC (#1 and 2) cell lines were analyzed (2-3 plates per cell line; 3–27 neuronal aggregates per plate). The loading solution consisted of OGB1 3.2 µM, Cremophor EL (Merck, Darmstadt, Germany) 0.01% v/v and Pluronic F-127 (Merck, Darmstadt, Germany) 0.4% in NDM. Cells in the loading solution were incubated for 1 h in the dark at 37°C and 7% CO_2_. After washing twice with NDM, the medium was replaced with ACSF, and cells were maintained for 30 min before imaging. The regions of interest were recorded for ∼4.7 min at 6.64 Hz (T = 0.1506 s; 1877 frames in total). TTX (0.2 µM) was added to the bath to confirm the AP-dependence of Ca^2+^ transients.

### Imaging analysis

#### Data pre-processing

Bleach correction was conducted for all recordings with the ImageJ Bleach Correction Macro Package (Miura, 2020). Contrast and brightness were adjusted manually using ImageJ tools. Motion correction and active neurons identification were performed with the CaImAn Constrained Nonnegative Matrix Factorization algorithm (Giovannucci et al., 2019). We will use the term “network” to refer to all the identified active neurons by CaImAn within the recorded visual field. Therefore, within each plate, several networks were recorded. Ca^2+^ transients (Figures 2C,D) in every active neuron were obtained by subtracting the neuron baseline fluorescence (quantile 8), and a fluorescence intensity matrix was built per network with rows corresponding to the different neurons and columns indicating the Ca^2+^ signal at every frame (point in time) during the recording time.

#### Network topology analysis

The Pearson’s correlation index between the signals of every pair of neurons in each network (rows in the fluorescence intensity matrix) was calculated. A FC matrix (with shape: neuron identity, neuron identity) was created with these correlations (Figure 2E). Two neurons were considered as connected if the absolute value of the correlation index was >0.4 (Figure 2F).

#### Frequency distribution of neuronal connectivity degree

For the Poisson and the Binomial fitting of the number of functional connections per neuron (connectivity degree; Figure 2G), we first built a histogram with binned connectivity degree data (11 bins). Then, the fitting was performed on the binned data with nonlinear least-squares regression using the *curve_fit()* function of SciPy python library, with *poisson.pmf()* for Poisson and *binom. pmf()* for Binomial fitting. For the power-law fitting (Figure 2H), the slope (scaling exponent) in each network was estimated by linearly regressing the logarithm (log_10_) of neurons connectivity degree on the logarithm of its frequency. The code employed to obtain the scaling exponents of each network is available at https://github.com/sofiapuvogelvittini/neuronal_functional_connectivity. Then, a linear mixed effect model, adjusting for the number of neurons and number of possible connections in the network (described in Eq. 1), was used to compare the scaling exponents between SZ and HC.

#### Identification of individual calcium transient events

Individual events were detected using TaroTools extensions (https://sites.google.com/site/tarotoolsregister/) implemented in Igor Pro (Wavemetrics, Lake Oswego, OR, United States). After a first automatic round using an amplitude threshold-based detection of 40% of the maximum amplitude for each trace, we visually confirmed the selection of events before subsequent analysis. The half-width was measured in 5 246 detected events and a histogram was built to visualize the distribution of individual events kinetics (Figure 3A.I)

#### Functional connectivity dynamics

To identify the different connectivity configurations occurring in the network during the recording period, we applied a sliding-time-window correlation method to the fluorescence intensity matrix (Cabral et al., 2017). This consists in calculating the Person’s correlation index for the signals of every pair of neurons in the network within a defined number of frames (width of the time-window). The window is then shifted in one step, to frame *t + 1,* repeating the procedure until all frames are covered (Figure 3A.II). Thereby, a FC matrix is obtained per step (wFC(t); Figure 3B.I). After measuring the kinetics of all detected calcium transients (see half-width histogram of Figure 3 A.I), we chose a width for the time-window of 70 frames (∼10.5 s), to capture the vast majority of different connectivity configurations while minimizing random correlations within the signal noise. To assess the sensitivity of our framework to changes in this parameter, we repeated the procedure using longer time-windows of 100 and 200 frames. As the wFC(t) matrices are symmetrical, we reshaped them into a one-dimensional vector containing only the values below the diagonal. FC dynamics matrices (FCD) were then obtained by computing the Pearson’s correlation coefficient between every pair of wFC(t) vectors (Figure 3B.II).

#### Functional connectivity meta-states

To quantify and compare the dynamics of FC in our networks, we adjusted a method designed by Miller et al. (2016) for fMRI data that allows both identification of discrete connectivity configurations and extraction of relevant features of FC dynamics. For each network, we conducted an independent component analysis (ICA) along the whole set of wFC(t) vectors, using the FastICA algorithm (Hyvärinen et al., 2000). Setting the same number of independent components across all the networks, we reduced the dimensions of all wFC(t) to a same number. The number of independent components was set to four, allowing algorithmic convergence in most recordings. Each wFC(t) vector was regressed on the four independent components or “correlation patterns” (CP; note that each network had its own specific CP basis) and the original vector was then described by the four corresponding regression coefficients or “weights”, each one associated with a CP (Figure 3B.III). Next, the weights were discretized into quartiles (Figure 3B.IV). As weights could be positive or negative, we treated them separately during the discretization process. We replaced CP weights by a value in ± (1,2,3,4), according to their signed quartile. A meta-state was then defined by a particular combination of four discretized weights and, while all networks presented the same number of potential meta-states, each network would typically visit a subset of them.

#### Functional connectivity-related variables

After obtaining the set of visited FC meta-states per network, we calculated the following FC-related variables describing the dynamics of the network connectivity:

- Number of visited meta-states: total number of different meta-states realized by each network.
- Number of change-points: number of transitions through the visited meta-states.
- Meantime in a meta-state: mean number of consecutive frames that the network remained in a meta-state, translated to units of time.
- Maximum distance between successive meta-states: maximum Manhattan distance (L1) between two consecutive meta-states.
- Traveled distance: sum of the distances between successively visited meta-states along the entire recording time.
- Dynamic range: Manhattan distance (L1) between the most distant visited meta-states.
- Number of hub meta-states: total number of different meta-states visited at least twice during the recording time.
- Meantime in a hub: mean number of consecutive frames that the network remained in the same hub meta-state, converted to units of time.
- The number of visits to hub states: number of times that the network visited any hub meta-state.

The number of frames was translated to units of time by multiplying it by the camera delay (∼0.1506 ms). The identification of meta-states and quantification of FC-related variables were performed using customized code written in Python, which can be found at https://github.com/sofiapuvogelvittini/neuronal_functional_connectivity. Since the FC-related variables were quantified in each different network, multiple measurements were obtained per cell line. Therefore, the “SZ effect” (diagnosis effect) was evaluated with a mixed linear regression model, including a random intercept for the cell line identity (id) to account for the internal variability within the different cell lines. As in most cases the FC-related variables correlated with the number of active neurons (see Results, Supplementary Figure S5B), we used deviance statistics to evaluate the necessity to adjust for the number of neurons and number of possible connections. In such cases, the variables were previously centered. We used the Statsmodels package of Python to fit the following model:

$$y\;∼\;\beta_{diagnosis}X_{diagnosis}+\beta_{\left(\#ofneurons\right)}X_{\left(\#ofneurons\right)}+\beta_{{\left(\#ofneurons\right)}^{2}}X_{{\left(\#ofneurons\right)}^{2}}+\left(1|cell\;line\;id\right)$$ (1)

*y* represents the dependent variable and can took the value of any FC-related variable, as well as the value of the scaling exponent obtained from the connectivity degree power-law fitting. *βi* are the coefficients associated with the *Xi* predictors. $X_{diagnosis}$ is a binary variable, coded as “1” for SZ neurons and as “0” for HC. Thus, the “SZ effect” corresponds to the value of *β_diagnosis_* and was considered statistically significant when its Z-score-associated *p*-value was <0.05. *β_diagnosis_* > 0 indicates a positive correlation of the variable *y* with the SZ condition, and the opposite for *β_diagnosis_* < 0.

More details and specifications of the regression models are presented in Supplementary Tables S3–S5.

## Results

### Molecular profiling of long-term hiPSCs-derived neuronal cultures from SZ and HC

**FIGURE 1:**
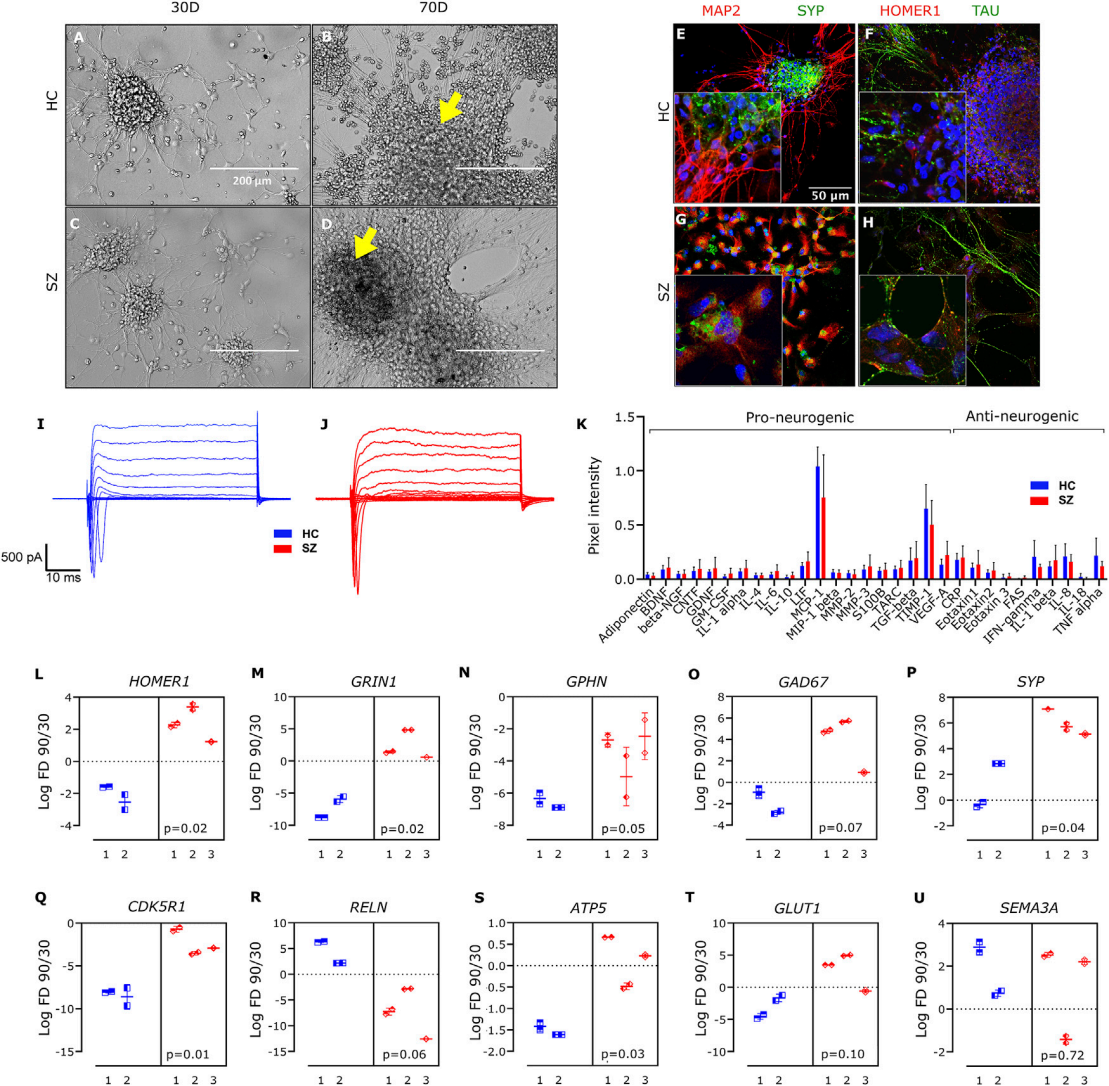
Profiling of hiPSCs-derived neurons obtained from HC and SZ patients. **(A–D)** Representative images for HC- **(A,B)** and SZ- **(C,D)** derived neuronal cultures, captured with phase-contrast microscopy at 30 **(A,C)** and 90 **(B,D)** days of differentiation. Arrows indicate 3D neuronal aggregates where spontaneous activity was recorded. Calibration bar: 200 µm. **(E–H)** Representative immune staining for the dendritic and axonal markers MAP2 **(E,G)** and TAU **(F,H)**, respectively, as well as for the pan-presynaptic and glutamatergic-postsynaptic proteins synaptophysin (SYP) **(E,G)** and HOMER1 **(F,H)**, respectively. DAPI staining is shown in blue. Calibration bar: 36 µm. **(I,J)** Representative voltage-clamp electrophysiological recordings at 60 days of differentiation, for HC and SZ-derived neurons. **(K)** Quantification of neurogenic proteins level (mean ± SD) in the CM of four SZ (#1–4) and three HC (#1–3) cell lines at 75 days of differentiation. **(L–U)** Fold-change in mRNA expression levels of different genes related to central nervous system development, in the period from 30 to 90 days. Data is presented as the log_2_ of the 90/30 days ratio for three SZ (#1, 2, and 3) and two HC (#2 and 3) cell lines, and every observation is reported independently. *B2M* was used as housekeeping. Excepting *SYP* in SZ#1, all measurements were performed in duplicates per cell line; *p*-value (p), from nested *t*-test.

Given the relative scarcity of studies on functional maturation in long-term hiPSCs-derived neuronal cultures, we first aimed to validate and characterize the differentiation process. We used hiPSCs-derived NSCs cultures from HC and SZ patients, displaying clear staining for NESTIN and PAX6, two well-known markers for NSCs (Casas et al., 2018). We modified the protocol of Shi et al. (2012) to induce long-term neuron-enriched cell cultures from four SZ and three HC NSCs lines (see Supplementary Table S1 for details). We omitted the use of antibiotics, as they may affect neuronal excitability (Bahrami and Janahmadi, 2013) and modify the expression of several genes (Ryu et al., 2017). HC and SZ patient-derived NSCs aggregated and formed neuronal rosettes that became larger and defined along the differentiation process (Izsak et al., 2019). After 30 days, 3D neuronal aggregates were already easily identified (Figures 1A–D), exhibiting unique structures that vary in size and shape within each culture plate. At 60 days, neurons expressed specific dendritic and axonal markers (MAP2 and TAU, respectively); MAP2 was expressed in the perikarya and dendrites (Figures 1E,G), while TAU was mainly expressed in axons (Figures 1F,H). Also, the expression of the pan-presynaptic vesicle-associated protein synaptophysin (SYP) (Figures 1E,G) and the postsynaptic scaffolding protein (HOMER1) (Figures 1F,H) confirmed the presence of synaptic structures. In addition, hiPSCs-derived neurons from both HC and SZ displayed voltage-dependent Na^+^ and K^+^ currents (Figures 1I,J). Consistent with Shi et al. (2012), at 100 days of differentiation we observed the presence of GFAP+ astrocytes, mostly in the surrounding area of the neuronal aggregates (Supplementary Figure S2).

The functional competence of our differentiated cell cultures was further assessed by analyzing their conditioned medium (CM). The expression profile of different cytokines and growth factors related to neuronal differentiation and signaling was evaluated in the serum-free CM, collected from SZ and HC cell lines (Figure 1K) at 75 days of differentiation. The complete set of targeted proteins was identified in the CM (although Eotaxin-3 and Interleukin-18 were barely detected; Supplementary Figure S3) of SZ and HC neurons, and quantification of the secreted proteins revealed a similar secretion profile between the two groups. Altogether, these results validate the correct establishment of HC and SZ mature neuronal cultures.

### Altered expression of genes involved in synaptic function and network establishment during SZ neurodevelopment

We analyzed the expression of a set of genes involved in synaptic function, cytoskeleton organization, and cellular metabolism in our neuronal cultures. Gene expression was quantified with qPCR at two different time points during the differentiation process: 30 and 90 days in culture (Supplementary Figures S1A,B). We evaluated genes encoding selected proteins expressed in glutamatergic synapses, including the glutamatergic NMDA-receptor subunit 1 (*GRIN1*) and *HOMER1* (Xiao et al., 1998); as well as genes encoding GABAergic synapse markers, such as the GABAergic postsynaptic anchoring protein (*GEPHYRIN*), associated with GABA receptors (Choii and Ko, 2015), and the glutamic acid decarboxylase 67 (*GAD67*), involved in the synthesis of GABA (Lau and Murthy, 2012). Also, we quantified the expression of *SYP*. SZ and HC neurons did not display significant disparities in the expression of the mentioned genes when compared at the same period of differentiation (Supplementary Figure S1B). To explore how potential glutamatergic and GABAergic synapses evolved over time in both conditions, we quantified the change in mRNA expression of the mentioned genes between 30 and 90 days *in-vitro* (90/30 days ratio; Figures 1L–P; for details on statistical tests and *p*-values, see Methods and Materials and Supplementary Table S2) and compared this change between SZ and HC networks. The change in *HOMER1* expression along differentiation was significantly different between both conditions (Figure 1L); while its expression decreased in HC, it increased in SZ (Supplementary Table S2). A difference between groups was also detected for *GRIN1*, with a significant decrease of *GRIN1* expression activity during differentiation in HC and no change in SZ (Figure 1M, Supplementary Table S2). These results suggest a divergent evolution in the number, or strength, of excitatory synapses during early neurodevelopment in SZ, as compared to HC networks. On the other hand, expression of the GABAergic marker *GPHN* was reduced in both conditions along this period of time (Supplementary Table S2), suggesting a decline in GABAergic communication during differentiation, and no difference was observed in *GPHN* expression change during the assessed period between SZ and HC neurons (Figure 1N). Also, no difference in *GAD67* expression change during differentiation was observed in SZ as compared to HC networks (Figure 1O). Conversely, the expression of *SYP* remained unaltered in HC neurons during the evaluated time period, whereas it increased in SZ networks (Figure 1P). Considering the increase in *HOMER1* and *GRIN1* expression in SZ networks during the differentiation process, the increase in *SYP* might reflect an increased number/activity of excitatory synapses in SZ, further supporting an altered evolution of synaptic connectivity over time in SZ. In this same line of evidence, a recent work using immunofluorescence staining indicated a higher density of SYP puncta in hiPSCs-derived neurons from patients with SZ (Yamamoto et al., 2021). Altogether, these observations support previous hypotheses proposing an altered excitatory/inhibitory balance in SZ (Allen et al., 2019), with a trend to develop hyperexcitability, and suggest that this imbalance may have its origin in the early stages of neurodevelopment.

We also evaluated expression changes during differentiation of *CDK5R1* and *RELN*, both involved in neurodevelopment. *CDK5R1* codes for p35, the neuronal-specific activator of cyclin-dependent kinase 5 (CDK5) regulatory subunit 1 (Ko et al., 2001). CDK5 contributes orienting neuronal network structure during neurodevelopment via cytoskeleton remodeling (Nguyen et al., 2002). Expression of p35, and hence CDK5 activity, varies cyclically along with brain development (Wu et al., 2000). During the assessed period, we found a reduction in *CDK5R1* expression in both conditions (Supplementary Table S2). Nonetheless, this decrement was significantly lower in SZ than in HC cultures (Figure 1Q).

The extracellular-matrix glycoprotein *RELN* plays a critical role in neuronal migration during early development (Wasser and Herz, 2017). Later in life, it modulates dendritic and axonal outgrow and spine maturation, by regulating cytoskeleton dynamics. In our neuronal cultures, the change in *RELN* expression between the 30 and 90 days of differentiation was similar for SZ and HC (Figure 1R).

Since metabolic anomalies were associated with SZ (Kumar et al., 2019), we also evaluated the expression of *ATP5*, coding for mitochondrial-membrane ATP synthase. *ATP5* expression decreased throughout the evaluated period in HC networks, but remained unchanged in SZ (Supplementary Figure S1, Supplementary Table S2). An abnormal energy metabolism during early neurodevelopment might have multiple consequences on network establishment and function (Hahn et al., 2020).

Finally, we did not detect changes in gene expression level of glucose transporter 1 (*GLUT1*), nor in the expression of the semaphorin family *SEMA3A*, along differentiation (Supplementary Table S2, Figures 1T–U).

### Calcium imaging in hiPSCs-derived neuronal networks

**FIGURE 2:**
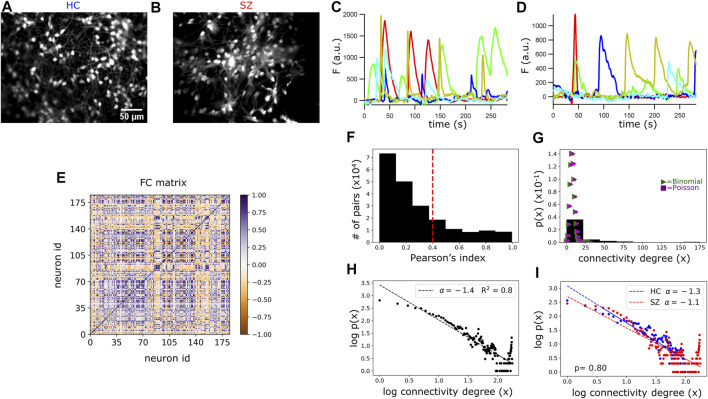
Functional connectivity (FC) and topology analysis in HC and SZ networks. **(A,B)** Representative ROI images of HC and SZ neuronal cultures loaded with the Ca^2+^ indicator OGB-1. **(C,D)** Ca^2+^ transients (fluorescence intensity after baseline correction) of 5 randomly selected neurons in a HC and a SZ network. **(E)** Representative static FC matrix displaying the Pearson’s correlation indexes (color bar) between the Ca^2+^ signals from each pair of neurons in a HC network, considering the whole recording time. **(F)** Frequency plot of the absolute Pearson’s correlation coefficients from every pair of neurons, considering all networks. The dotted red line indicates the threshold beyond which we considered a pair of neurons as functionally connected. **(G)** Probability density of finding a neuron with x functional connections (connectivity degree). The connectivity distribution does not fit to a Poisson (λ = 8) nor to a Binomial (n = 1,000, *p* = 0.008) distribution. **(H)** Scatter plot of the connectivity degree logarithm (log_10_) vs. the probability logarithm. Data fit a scale-free or long-tailed distribution, with a scaling exponent of −1.4. **(I)** Scale-free fitting of neurons connectivity degree in SZ and HC networks. Connectivity scaling exponents were fitted with a mixed effect regression model detailed in Methods. The scaling exponents did not significantly differ between the two groups. *p*-value (p).

Spontaneous spiking activity of hiPSCs-derived populations of neurons was registered at single cell resolution, by monitoring changes in intracellular Ca^2+^ concentration (Ali and Kwan, 2020). Cell cultures were loaded with the cell-permeant version of the Ca^2+^ indicator OGB1 (Figures 2A,B) and 4.7 min length videos (1877 frames per video; T = 0.1506 s) were recorded across different regions of interest (ROIs) within the cell plates. As previously described (Izsak et al., 2019), higher neuronal density and spontaneous activity was more likely to be found in cell aggregates protruding from the base of the plate (Supplementary Figure S4 and yellow arrows in Figures 1B,D). Therefore, we considered them as the ROIs to study local neuronal network activity. We identified the active neurons within these ROIs using the open-source tool CaImAn software, considering a signal-to-noise ratio above 2.5 (Giovannucci et al., 2019), and the set of active neurons in the aggregate was defined as a neuronal network (134 networks in total, 3–27 networks per plate, 2-3 plates per cell line, 3 SZ and 2 HC cell lines). Then, Ca^2+^ transients were obtained from every neuron in the network (Figures 2C,D). The number of active neurons within each network varied from 15 to 250; however, regression analysis revealed no difference in the mean number of active neurons per network between SZ and HC (Supplementary Figure S5A, mixed regression model detailed in Methods Eq. 1).

Voltage-gated Na^+^ channels are responsible for the initiation and propagation of action potentials in neurons (Noebels et al., 2012). To probe the action-potential dependency of the spontaneous Ca^2+^ events observed in the networks, we added the selective Na^+^ channel blocker toxin tetrodotoxin (TTX; 0.2 µM) to the bath during recordings. Ca^2+^ transients were abolished by the blockage of the voltage-gated Na^+^ channels (Supplementary Figure S6), corroborating the neuronal origin of the detected activity.

### Network topology analysis of hiPSCs-derived neuronal cultures based on functional connectivity measurements

Previous studies assessed neuronal structural connectivity in hiPSCs-derived neurons using trans-neuronal spread of rabies virus (Brennand et al., 2011; Kirwan et al., 2015). Neuronal cultures derived from SZ patients presented a decrease in trans-synaptic tracing (Brennand et al., 2011), which might be related to a decrement in the number of synaptic connections. However, contrary to this hypothesis, electrophysiological recordings indicated similar total spontaneous synaptic activity between SZ and HC neuronal cultures (Brennand et al., 2011). In the present work, we addressed neuronal network connectivity from a functional perspective by quantifying the co-variation in neurons activity patterns, registered with Ca^2+^ imaging, in SZ and HC. Since the underlying network topology frames neuronal interactions, assessing FC in this *in-vitro* system represents both an indirect approach for revealing the structure of the networks and a direct way to interrogate their emerging functional properties.

To quantify neurons FC, we calculated the Pearson’s correlation index for the Ca^2+^ signals from each pair of neurons in every network. As a first approach, we considered the entire recording time (4.7 min) and generated a FC matrix (or correlation matrix) displaying the correlation indexes between each pair of neurons (Figure 2E). Then, the number of functional connections per neuron was obtained from this FC matrix, by considering a pair of neurons as functionally connected when the correlation index was above 0.4 or below −0.4. For an absolute correlation value of 0.4 as a cut-off point, the pairs of functionally connected neurons reached 25% of the total possible connections, considering all networks (Figure 2F). If the total number of connections per neuron was randomly determined, the probability for a neuron to have “x” connections (connectivity degree) would follow a binomial distribution and for large enough populations, a Poisson distribution (Figure 2G). In contrast, the probability distribution for the number of connections per neuron was right-skewed, resembling a “scale-free” distribution (Figure 2H; also known as “long-tailed” or “power-law” distribution). This suggests that a large number of neurons had few active connections, while a small number were hyper-connected (Figures 2G–I). Interestingly, this connectivity organization has been proposed for the CNS across different species, including humans (Bullmore and Sporns, 2009; Zhang et al., 2021), and our results add evidence suggesting that it might be conserved in early neurodevelopment.

The scale-free fitting of neurons connectivity degree followed a power-law with a scaling exponent of −1.4 (Figure 2H). Using trans-neuronal spread of rabies virus, a previous analysis of network topology in hiPSCs-derived neurons demonstrated that the number of structural connections of the neurons also followed a scale-free distribution, with power -2 (Kirwan et al., 2015). Taken together, these observations support the suitability to explore topological properties of hiPSCs-derived neuronal networks through FC analysis, by establishing a link between structural and functional communication.

To test for differences in the functional topology between SZ and HC, we fitted a mixed linear model for the networks power-law scaling exponents, including a varying random intercept for cell line id and adjusting for the number of neurons and number of total possible connections in each network (see Methods Eq. 1). The functional topology of SZ networks did not deviate significantly from the HC networks topology (Figure 2I).

### Functional connectivity in hiPSCs-derived neuronal networks is dynamic and goes through reoccurring configurations

The FC analysis of resting networks described so far represents the average connectivity among pairs of neurons along the entire recorded time (4.7 min). However, this approach does not allow to explore dynamic fluctuations of connectivity that could occur within shorter periods of time.

**FIGURE 3:**
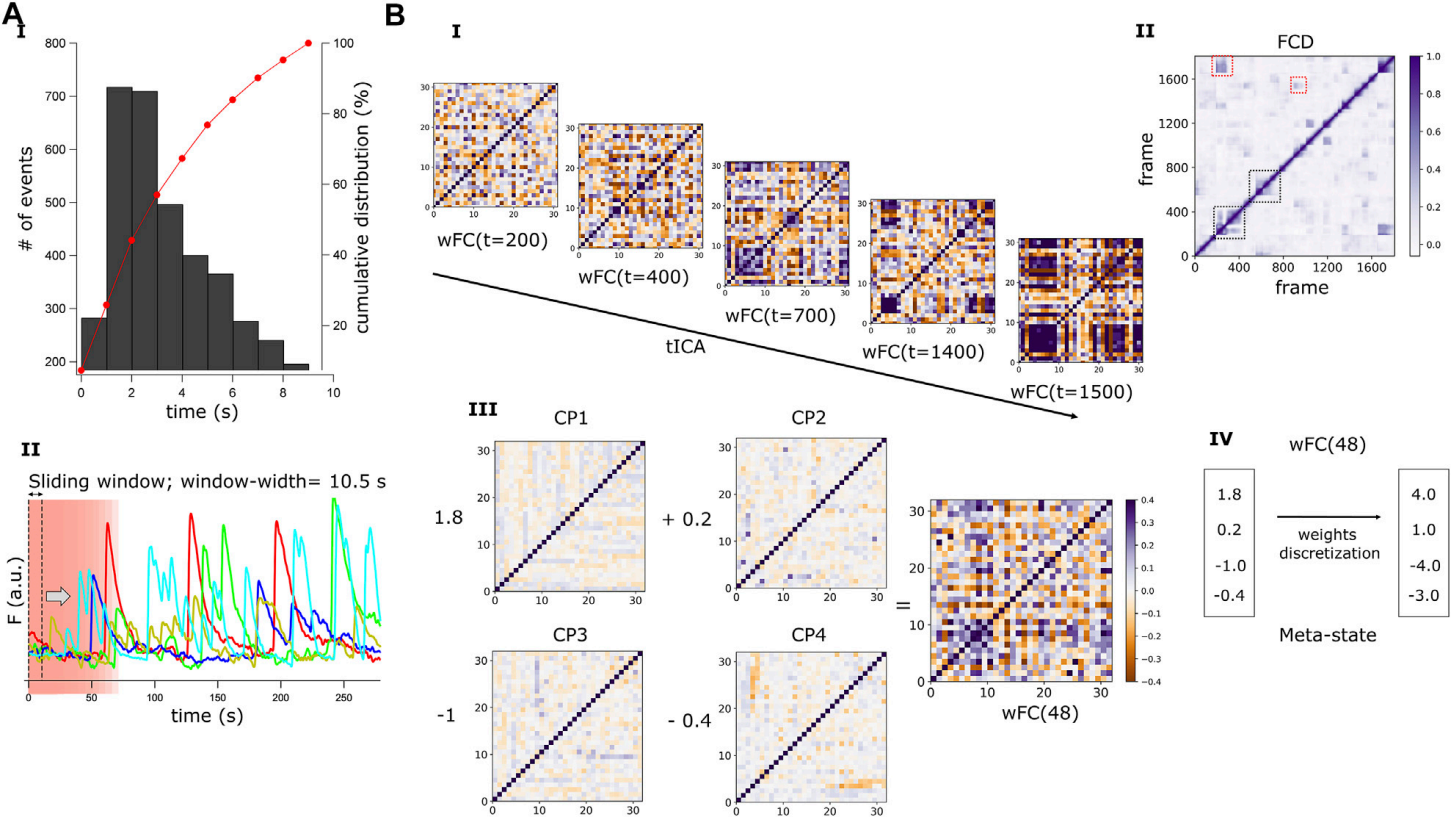
Dynamics of the resting-state FC and definition of connectivity Meta-states. **(A)** Graphical representation of the sliding time-window correlation method. **(A.I)** Histogram showing the distribution of the transients half-widths for all detected Ca^2+^ events (n = 5,246). The cumulative distribution (red curve) depicts that no events with a half-width longer than 10s were detected. **(A.II)** Ca^2+^ signals of 5 randomly selected neurons in a HC network composed of 32 active neurons. A sliding time-window of width 10.5 s (70 frames) is represented by a pink rectangle, within which the correlation between the signals originating from each pair of neurons is calculated. The time-window scrolls through the entire recording time (1877 frames), advancing one frame at each step, and a windowed correlation matrix (wFC) of shape number of neurons, number of neurons, is obtained at each step (t). **(B)** Windowed correlation matrices and definition of Meta-states. **(B.I)** A wFC(t) is obtained for each time step (t), with *t* running from 1 to 1808. **(B.II)** A representative Functional Connectivity Dynamics matrix (FCD) obtained from a HC network. This time versus time FCD matrix is obtained by calculating the Pearson’s coefficient between every pair of wFC(t) in the network. Periods of lasting FC patterns are reflected by square blocks around the FCD diagonal (black dotted squares) and reoccurring patterns by square blocks distant from the diagonal (red dotted squares). **(B.III)** For every recorded network, an independent component analysis (tICA) is performed along the temporal axis of the whole set of wFC(t), and four independent connectivity patterns (CP) are obtained. Then, each wFC(t) can be described as a linear combination of these four CP and **(B.III)** shows wFC(48) as an example. The coefficients (weights) multiplying each CP are obtained by linearly regressing wFC(48) on the CPs. **(B.IV)** To have a finite number of states (meta-states, MSs), the weights associated with the CPs are discretized and assigned to their respective quartile. The combination of these four discretized coefficients describes a MS.

The temporal dimension of the FC in the hiPSCs-derived networks was addressed with a sliding-time-window correlation method (Cabral et al., 2017) (Figure 3A). To choose an appropriate width for the time-window, we first built a histogram depicting the distribution of the half-width of all detected Ca^2+^ events (n = 5,246; Figure 3A.I). As no events with a half-width longer than 10 s were detected, we used a sliding time window of width = 10.5 s (70 frames, Figure 3A.II) to capture most of the different connectivity configurations while minimizing random correlations within the signal noise. For each network, we obtained a set of windowed FC matrices (wFC(t); where *t* is the time step, running from 1 to 1808) (Figure 3B.I). Then, we computed the correlation matrix for every pair of wFC(t), obtaining a time versus time functional connectivity dynamics matrix (FCD) (Figure 3B.II). The FCD diagonal results from comparing the same time points, thus displaying full correlation. Points located adjacent to the diagonal (Figure 3B.II, black dotted squares) are also expected to present high correlation, since they correspond to nearby, partially overlapping, time-windows. However, the squared blocks far away from the diagonal (Figure 3B.II, red dotted squares) suggest the reappearance of previously visited FC configurations in a distant time point. This reveals that, during the recording time, the network resting-state FC went through numerous and reoccurring configurations, rather than evolving in an arbitrary way. Remarkably, this observation resembles previously described FC dynamics in the human brain at rest (Hutchison et al., 2013).

Next, we developed a quantitative framework to evaluate and compare the dynamics of FC between SZ and HC networks, by modifying a previously published method that was designed to evaluate whole brain functional connectivity dynamics in patients with SZ (Miller et al., 2016). To reduce the dimensions of the wFC(t) matrices, obtained from the different networks, to the same number of dimensions (note that the dimensions of the wFC matrices depend on the number of active neurons in the particular network), we ran an independent component analysis (ICA) (Hyvärinen et al., 2000) per network, throughout the temporal dimension of its FC [Figure 3B.I, tICA on the set of wFC(t)]. This computational method allows the separation of a complex signal into additive components (Hyvärinen et al., 2000). Four different and independent FC configurations, defined here as *correlation patterns* (CP), were identified per network. To describe each of the wFC(t) in terms of these four CP, we obtained four coefficients or “weights” by linearly regressing every wFC(t) on the CPs (Figure 3B.III). Then, to capture a finite number of FC configurations and to define the same maximum number of different FC states across all the networks, the weights were discretized into quartiles, allowing similar connectivity configurations to be grouped together (Figure 3B.IV). The unique combination of four discretized weights defined a Meta-state (MS), borrowing the term coined by Dr. Robyn Miller (Miller et al., 2016).

### Resting-state functional connectivity in SZ networks is less flexible and slower in rearranging different configurations compared to HC

**FIGURE 4:**
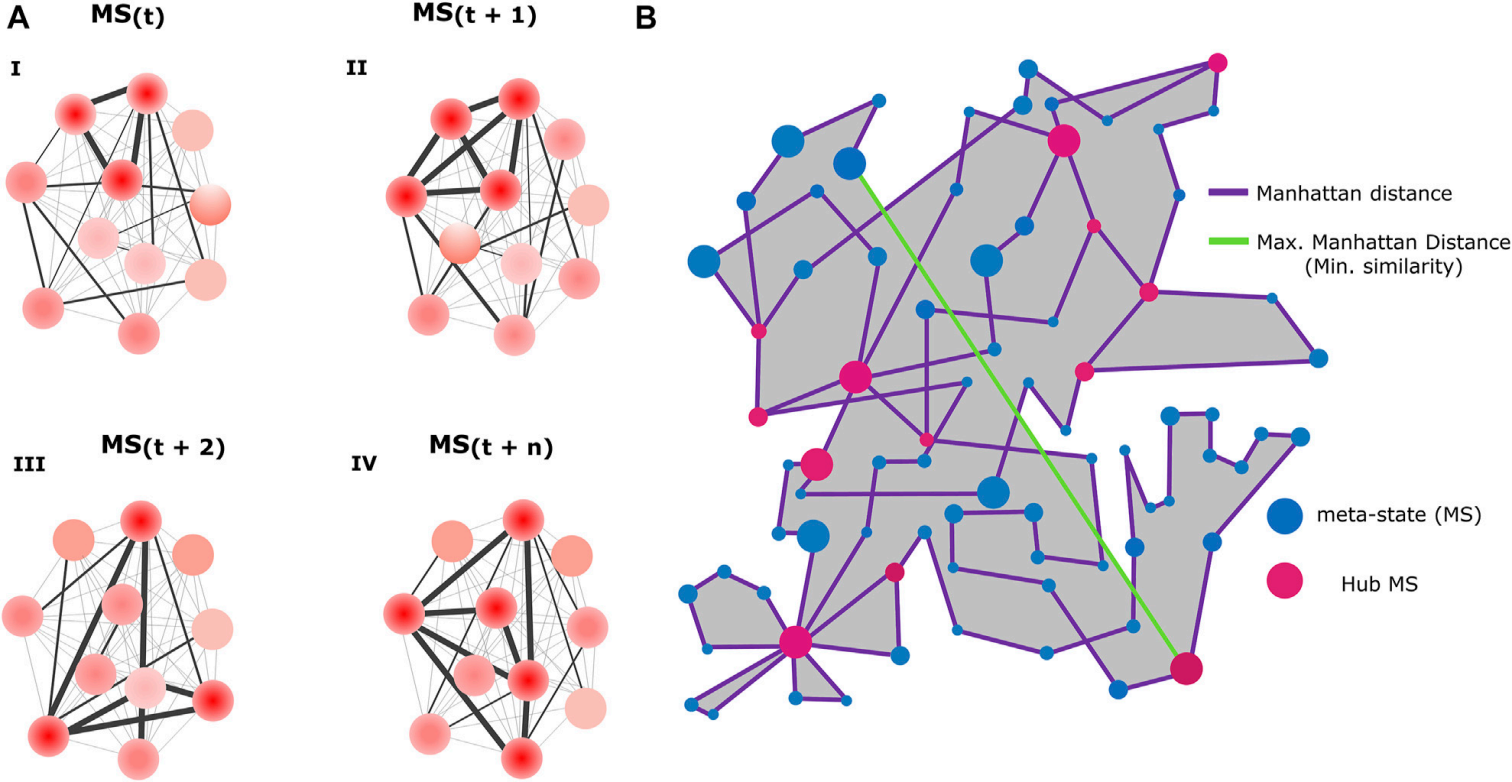
Functional connectivity-related variables. **(A)** Schematics represent four possible functional connectivity meta-states (MSs), corresponding to particular activity configurations of the neurons conforming the network. Each circle represents a neuron and color intensity the strength of its activity (low red = low activity, high red = high activity). The thickness of the straight lines connecting the neurons reflects the strength of the correlation between the signals emitted by the neurons (thicker = higher correlation). **(B)** Diagram illustrating an example of the resting-state FC flow of a network, with a description of all measured FC-related variables. Blue circles represent the different visited MSs. Each MS inhabits a 4-dimensional space, determined by the 4 weights associated with the independent connectivity patterns. However, for visualization purposes, in this scheme each MS is represented in a 2-dimensional space with arbitrary units. Circle size denotes the uninterrupted time (dwell time) spent by the network in each configuration. Purple lines represent successive transitions between different MSs. The length of the lines corresponds to the Manhattan distance between two connected MSs (in the space where the MSs reside) and reflects how different are successive MSs, with longer lines indicating higher dissimilarity. As a measure of network flexibility, we evaluated the ability of SZ and HC networks to switch in just one step between two configurations that are as different as possible, a transition represented by the maximum distance achieved between two successive MSs (green line). The pink circles represent recurring MSs (Hub MSs), which are connected by more than two lines, meaning that the network visited them at least two times. The grey area corresponds to the dynamic range, a global indicator of the potential diversity of accessible MSs. The length of the trajectory of the purple line corresponds to the total traveled distance. Panels **(A and B)** were not generated from real data and constitute solely graphical illustrations.

By studying neuronal functional relationships in our *in-vitro* model, we aimed to test whether some features of the resting-state FC dynamics observed in SZ patients (Miller et al., 2016) might also be observed in neural networks resembling early neurodevelopmental stages in SZ. Using the methodology described in Figure 3, we obtained the FC MSs visited by each network and quantified a set of FC-related variables (listed and defined in Methods) in both conditions. Figure 4 aims to clarify the meaning of these variables, with Figure 4A illustrating examples of different possible MSs visited by a network and Figure 4B including a graphical explanation of the measured variables.

Differences in FC-related variables between SZ and HC networks were assessed using mixed linear regression modeling. Even though the average number of active neurons did not differ between SZ and HC networks (Supplementary Figure S5A), in most cases the measured FC-related variables correlated with it (Supplementary Figure S5B). Therefore, we included the number of active neurons as a covariable in almost all the multiple regressions (Supplementary Table S3).

**FIGURE 5:**
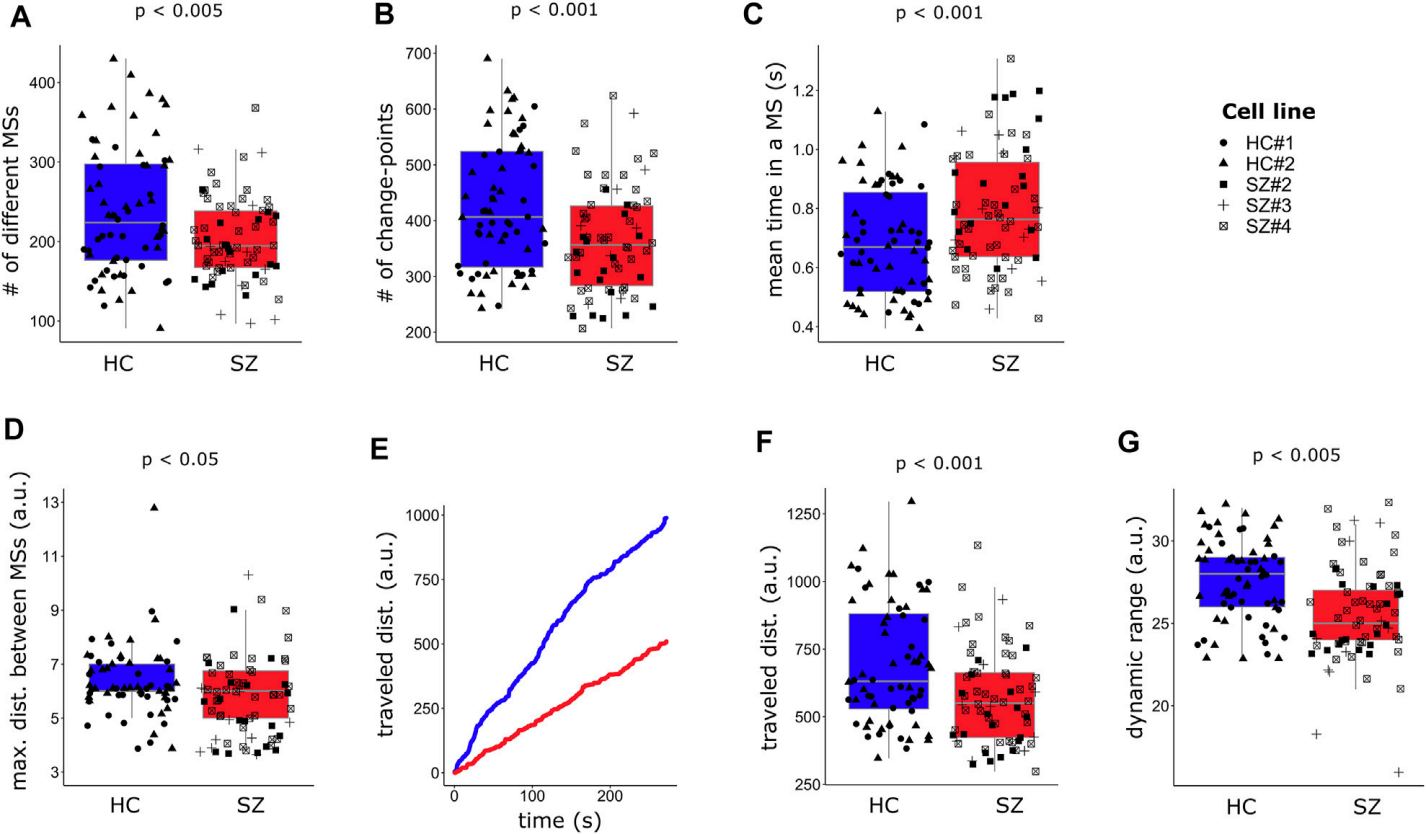
FC in SZ networks is less dynamic than in HC. **(A)** SZ networks visit a lower number of different meta-states (MSs) and **(B)** change fewer times from one MS to another during the recorded time, spending longer uninterrupted time at the same MS **(C)**. **(D)** The maximum distance, Manhattan distance (L1), between successive MSs is reduced in SZ neuronal networks. **(E)** A graphical representation of how FC travels within the space inhabited by the MSs, in terms of the L1 distance between successive visited MSs. The plot indicates the cumulative traveled distance, measured as the total traveled distance at each time point. **(F)** The overall distance traveled through the MSs space, calculated as the sum of all L1 distances across the visited MSs, is reduced in SZ networks. **(G)** The intrinsic dynamic range, measured as the L1 distance between the most distant MSs, is also reduced in SZ networks. Each single data is plotted independently on the boxplots and its symbol indicates the cell line of the particular network. For each dependent variable (*Y*-axis), the *p*-value (*p*) associated with the effect of the diagnosis from the regression model (Supplementary Table S3) is indicated. A time-window of width = 70 frames (∼10.5 s) was used for capturing the visited MSs.

We observed a reduction in the number of different MSs visited by SZ networks (Figure 5A), suggesting a more limited repertoire of accessible configurations in SZ, as compared to HC networks. We also measured the number of times that networks changed from one MS to another (Figure 5B), and the mean uninterrupted time spent in an MS (Figure 5C). SZ networks switched fewer times from one MS to another and remained longer at the same MS, evidencing a reduced dynamism. In addition, HC networks were capable to transit to more different (distant) configurations in one step (maximum distance between successive MSs, Figure 5D) than SZ networks, suggesting that SZ networks have less potential to fast switching between distant FC configurations, reflecting reduced flexibility. We also calculated the total traveled distance within the MSs inhabited space, defined as the sum of the distances between successive MSs through the whole recorded time (Figure 5E), finding that it was reduced in SZ (Figure 5F). Finally, we evaluated the dynamic range by calculating the Manhattan distance between the farthest MSs visited along the whole trajectory, which is formally a measure of the size of the space containing the different visited MSs (Figure 5G). We must bear in mind that the dynamic range is relative to the specific CPs of each network. Therefore, this measurement reflects how much the space where the different MSs inhabit is exploited with respect to the network intrinsic “motion possibilities” and can be interpreted as the potential of the network to flow through diverse connectivity configurations. We observed a reduced FC dynamic range in SZ networks compared to HC (Figure 5G), which is consistent with the lower number of different visited MSs and the reduced total traveled distance in SZ networks, since a lower dynamic range would imply a decrease in FC motion potential.

Considering that the value of the FC-related variables might be sensitive to the width of the time-window, used to capture the visited MSs, we repeated the complete procedure for obtaining the MSs using two different time-windows of widths 100 and 200 frames (15.1 and 30.1 s, respectively), and compared the estimated FC-related variables between SZ and HC. The results of these analyses are depicted in Supplementary Tables S4, S5. All the results were replicated for larger time-window widths (Supplementary Tables S4, S5).

Summarizing, our observations suggest that resting-state SZ networks have fewer available FC configurations, explore them less thoroughly and reorganize their activity patterns less rapidly as compared to HC networks. Overall, these findings point to a reduction in FC dynamism and flexibility in SZ networks, involving speed and exploration potential.

### SZ networks display a reduced number of recurrent functional connectivity meta-states (hub MSs) as compared to HC networks

**FIGURE 6:**
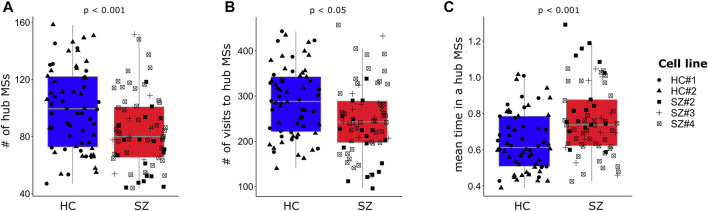
SZ networks visit a lower number of different hub MSs but remain longer uninterrupted time in them. **(A)** The number of different visited hub MSs is lower in SZ networks. **(B)** SZ networks visit hub MSs less often than HC networks, but **(C)** spend longer uninterrupted time in the same hub MS. Each single data is plotted independently on the boxplots and its symbol indicates the cell line of the particular network. For each dependent variable (*Y*-axis), the *p*-value (*p*) associated with the SZ effect from the regression model (Supplementary Table S3) is indicated. A time-window of width = 70 frames (∼10.5 s) was used for capturing the visited hub MSs.

As mentioned before, neuronal networks went through different FC configurations that reoccurred during the acquisition time (Figure 3B.II, FCD matrix). Therefore, we quantified the number of different MSs that were revisited by each neuronal network. In whole brain fMRI FC analysis, these recurrent MSs have been named as “hub meta-sates” (Miller et al., 2016), and here we named them in the same way. SZ networks displayed a lower number of different hub MSs (Figure 6A) and visited these hub MSs less often than HC networks (Figure 6B), but spent longer uninterrupted time in them (Figure 6C). These results were replicated when a larger time-window width of 100 frames (15.06 s) was used for obtaining the visited MSs (Supplementary Table S4). However, the differences between SZ and HC networks in terms of the number of different visited hub MSs and the number of visits to hub MSs were no longer observed for a time-window width of 200 frames (Supplementary Table S5).

## Discussion

Cognitive functions require the integration of neural activity through different scales, from neurons and local circuits to large-scale brain networks. Considering the evidence pointing to FC alterations in patients with SZ (Garrity et al., 2007; Whitfield-Gabrieli et al., 2009; Miller et al., 2016; Sheffield and Barch, 2016; Erdeniz et al., 2017), this study aimed to evaluate whether FC differences between SZ patients and HC subjects could be explored during early nervous system development *in-vitro,* using hiPSCs-derived neuronal networks. Our data indicate that FC in SZ neuronal networks is characterized by a narrower diversity of connectivity configurations, impaired dynamism and lower flexibility, compromising the ability of the networks for a rapid and efficient reorganization of their activity patterns. Remarkably, the altered FC dynamics in our hiPSCs-derived neuronal networks followed the same direction as the resting-state connectivity dynamics observed in the brain of patients with SZ (Miller et al., 2016).

We observed that resting-state FC in long-term neuronal cultures is a dynamic but not random phenomenon that exhibits recurrent configurations, resembling what has been described in the human brain using fMRI, which characterizes by a much lower spatial and temporal resolution as compared to our *in-vitro* methodology (Cabral et al., 2017). This observation is consistent with the concept of a “default mode” of neural activity, detected in different brain areas in resting condition, which may contribute to a basal activity state that provides an adequate starting point for efficient response to environmental changes (Raichle and Snyder, 2007). Hence, addressing the differences and similarities of activity configuration dynamics in resting-state across different spatiotemporal domains, could contribute to understanding the neurobiological bases of brain integration in both health and disease. With this in mind, we explored possible fundamental differences associated with SZ in the dynamics of neuronal network auto-organization, by evaluating FC-related variables in hiPSCs-derived cultures resembling early neurodevelopment. According to our results, SZ networks visited fewer different FC meta-states (MSs), exhibited a reduced number of transitions and spent longer uninterrupted periods of time in the same connectivity configurations, as compared to HC. In addition, the maximum change in a single step that the FC configuration could achieve was reduced in SZ networks, evidencing alterations in FC fast switching associated with SZ. Also, the decreased dynamic range observed in SZ networks suggests that the possibility of different FC configurations in SZ neuronal networks is more restricted than in HC circuits, which is consistent with the lower number of different MSs visited by SZ networks. These early developmental alterations in neural communicational dynamics described in SZ might be jeopardizing the ability of the nervous system for fast and efficient reorganization and converging into a more vulnerable organism to harmful or stressful external factors, leveraging it to SZ development during adulthood (Maynard et al., 2001).

An important observation was the presence of recurrently visited FC MSs (hub MSs) in hiPSCs neuronal networks. SZ networks showed a reduced repertoire of hub MSs and visited hub MSs less frequently, but remained longer periods of time trapped at the same hub configuration. It has been proposed that long-term potentiation (LTP; a widely studied model of synaptic plasticity) could constitute the neurophysiological basis for the formation of brain hub states (Stampanoni Bassi et al., 2019). The reduction in the number of hub MSs in SZ networks might be related to an altered plasticity mechanism for reinforcing certain neuronal connections during neurodevelopment, resulting in fewer different configurations strengthened in SZ networks. Actually, genome wide-association studies (Kirov et al., 2012; Fromer et al., 2014; Purcell et al., 2014) and a recent single-nucleus RNA sequencing analysis of SZ *post-mortem* brain tissue (Batiuk et al., 2020), evidenced alterations in the sequence and expression of genes involved in signaling pathways related to plasticity in SZ (Hall et al., 2015).

Interestingly, the reduction in the number of different hub MSs and total visits to hub MSs displayed by SZ networks, was no longer detected when increasing the width of the time-window to ∼30 s (200 frames). An overall decrease in the number of visited MSs was observed while the time-window width was extended (Supplementary Tables S3–S5). A plausible interpretation of these results may be a progressive reduction in the ability to discriminate some populations of visited MSs when using longer time windows. Presumably, the MSs that could no longer be detected were short-lived connectivity configurations. In this line, a possible explanation for the loss of statistical differences in the number of hub MSs and total visits to hub MSs between SZ and HC, when using the longest time-window, could be that SZ networks have a lower proportion of short-lasting hub MSs compared to HC. Thereby, the percentage of missed events would be smaller in SZ networks as compared to HC when using the longest time-window, which might reduce the differences in the number and total visits to hub MSs between the groups. This possibility is consistent with the reduced dynamism of FC in SZ networks. Overall, these results highlight the relevance of improving the time resolution for acquisition and analysis of FC data.

Several genes related to neurodevelopment and synaptic function have been implicated in the pathophysiology of SZ [discussed in (Birnbaum and Weinberger, 2017)]. We observed that the expression of genes encoding key glutamatergic postsynaptic proteins presented an altered evolution during early development in SZ networks. The decreased expression of excitatory synaptic components in HC neurons along with differentiation contrasted with the rising trend observed in SZ, whereas the expression of the gene encoding the GABAergic synaptic scaffold *GEPHYRIN* similarly decreased in SZ and in HC neurons during differentiation. Taken together, these results suggest an impairment in the excitation/inhibition balance in SZ networks during early development, with a possible trend to develop hyperexcitability. This is consistent with previous observations in patients with SZ (Whitfield-Gabrieli et al., 2009; Allen et al., 2019; McHugo et al., 2019) and also with transcriptomic analysis of cerebral organoids derived from SZ patients (Kathuria et al., 2020). It could be hypothesized that a shift in the excitation/inhibition balance producing neuronal hyperexcitability might cause the network to approach saturating levels of activity, which might lead to a more restricted set of possible connectivity configurations. This would be consistent with the lower network dynamism observed in SZ networks. However, as we only evaluated the expression of a few synaptic genes, we cannot establish an overall view of the number/activity of potential inhibitory and excitatory synapses in the networks. Instead, we can only estimate, according to the genes we evaluated, that there seems to be a tendency to hyperexcitability in SZ networks.

The observed difference in *ATP5* expression pattern between SZ and HC neurons during differentiation might reflect alterations in the cellular metabolism during early developmental stages in SZ. Interestingly, Hahn et al. (2020) described the intimate relationship between brain metabolic demands and FC during cognitive tasks. It would have been informative to evaluate relationships between network gene expression and the measured FC-related variables; however, to collect enough material to quantify gene expression, we pooled cell cultures from different plates. Therefore, we cannot relate gene expression with functional characterization derived from the same plate.

The expression and activity of CDKR5, and so the activity of CDK5, is tightly regulated and cyclically varies during development and adulthood (Delalle et al., 1997; Nguyen et al., 2002). According to our data, the reduction in *CDK5R1* expression during neuronal differentiation was less pronounced in SZ networks as compared to HC, contrasting with *post-mortem* examinations of brain tissue from SZ patients indicating reduced expression of *CDK5R1*, as compared to control tissue (Engmann et al., 2011; Ramos-Miguel et al., 2013). Together, these findings may indicate a dysregulation in the upstream molecular mechanisms that control *CDK5R1* expression in SZ, leading to divergences in *CDK5R1* expression throughout development and adulthood.

We are aware that additional factors might be influencing our results and thus it is necessary to be cautious in their interpretation. The main limitation is perhaps the low number of cell lines and replicates used to generate the data. In addition, the incomplete information available on the life history of the donors could be misleading the interpretation of our results, since other covariates might contribute to the differences between the groups. Future experiments including larger samples are crucial to validate our observations in SZ. A technical limitation of our methodology is the arbitrary definition of the focus during calcium imaging. As our cultures were composed of multiple cellular layers of overlapping neurons, the focus election defined the spatial limits of the analyzed networks, which may have influenced the resulting MSs and the inference of FC-related variables.

FC in neuronal cultures has also been evaluated using extracellular microelectrode arrays (MEAs). MEAs allow monitoring of electrical activity at many sites of a network. Nowadays, the development of high-density MEAs allows discrimination of electric signals near the single-cell resolution in cellular cultures (Poli et al., 2015). In addition, MEAs recordings exhibit higher temporal resolution than calcium imaging, allowing more precise delineation of the activity transients, and thereby, a more precise estimation of FC. Our analysis designed to quantify FC dynamics could be applied in MEAs data; thereby, the focus limitation inherent to calcium imaging would be improved and the increase in temporal resolution may contribute to identify short-lived connectivity configurations. Furthermore, due to the high temporal resolution achieved with MEAs, point-processes, such as spike trains, can be estimated from the time-serial records obtained with this technique. In that case, the directionality of FC could be estimated with a cross-correlation analysis, which would allow identifying a greater diversity of connectivity MSs by discriminating between neurons acting either as effectors or receptors of the activity.

Regarding the functional topology of the networks, positive as well as negative correlations were identified, suggesting the influence of excitatory and inhibitory synapses on the diversity of networks connectivity patterns. This was supported by the expression of excitatory (*HOMER1* and *GRIN1*), as well as inhibitory (*GPHN* and *GAD67*) synapses marker genes in the networks. However, the slow time course of the calcium transients makes the interpretation of the negative correlation indexes not straightforward, due to possible mono and multi-synaptic influences. Here, we used a similar differentiation procedure as Kirwan et al., 2012 (Kirwan et al., 2015), who showed that while GABA-A and B receptors are functional, application of a GABA-A receptor antagonist had no effect on the highly synchronized activity, indicating no significant influence of inhibition on this form of coordinated activity. However, authors did not check the effect of GABA inhibitors in a form of recurrent activity where massive synchronization was less frequent or absent (Kirwan et al., 2015), as was the activity presented in our networks. Therefore, the effective participation of inhibitory synapses in the generation of negative correlations in our networks cannot be ruled out and requires further investigation.

Collectively, our findings support previous hypotheses proposing that the brain is a complex system that may possess spatiotemporal scale-invariant principles governing its structure and function through development (Witvliet et al., 2021) [discussed in (Bullmore and Sporns, 2009; Zhang et al., 2021)], and add evidence suggesting that these principles are expressed at very early stages of development, when neuronal networks are in an emerging state. Of note, we were able to detect aberrant neural communicational dynamics already in the developing neuronal networks of SZ patients that may contribute to the altered FC described in the SZ brain. The integration of our methodology, aiming to evaluate network functional performance, with genome-wide transcriptomic analysis may further contribute to understanding the molecular and cellular mechanisms underlying FC impairments in SZ.

## Data Availability

The original contributions presented in the study are included in the article/Supplementary Materials, further inquiries can be directed to the corresponding authors. The code is available at https://github.com/sofiapuvogelvittini/neuronal_functional_connectivity.
