# Supplementary material for: Altered resting-state functional connectivity in hiPSCs-derived neuronal networks from schizophrenia patients
Source: Front Cell Dev Biol. 2022 Sep 7;10:935360. doi: 10.3389/fcell.2022.935360 (PMC9489842; doi:10.3389/fcell.2022.935360)
Supplement: Supplementary file 1 [file DataSheet1.docx]

**SUPPLEMENTAL INFORMATION**

**Table S1. hiPSCs lines used in this study.**

| **Code** | **cell line** | **diagnosis** | **sex** | **age (range)** | **cell Source** | **reprogramming Technique** |
| --- | --- | --- | --- | --- | --- | --- |
| HC#1 | GM23279A | Control | F | 36-40 | Fibroblast | Cytotune 1.0 kit (ThermoFisher) |
| HC#2 | CF2 | Control | M | 31-35 | Fibroblast | Cytotune 1.0 kit (ThermoFisher) |
| HC#3 | ADHD2 | Control | M | 31-35 | Urine Endothelial | (OCT4, SOX2, KLF4, MYC) |
| SZ#1 | GM23760B | Schizophrenia | M | 26-30 | Fibroblast | Cytotune 2.0 kit (ThermoFisher) |
| SZ#2 | GM23761B | Schizophrenia | F | 26-30 | Fibroblast | (OCT4, SOX2, KLF4, MYC, LIN28) |
| SZ#3 | EZQ3 | Schizophrenia | M | 41-45 | Fibroblast | Cytotune 2.0 kit (ThermoFisher) |
| SZ#4 | EZQ4 | Schizophrenia | M | 41-45 | Fibroblast | (OCT4, SOX2, KLF4, MYC, LIN28) |

Information about diagnosis, sex and age of the patients [1, 2]

**Figure S1. qPCR in neuronal cultures at 30 and 70-91 days of differentiation.**

(A) Primers used for qPCR amplification. mRNA expression levels of different genes related to nervous system development at 30 (B) and 70-91(C) days of differentiation. *B2M* was used as housekeeping gene. Data are shown as mean ± SD.


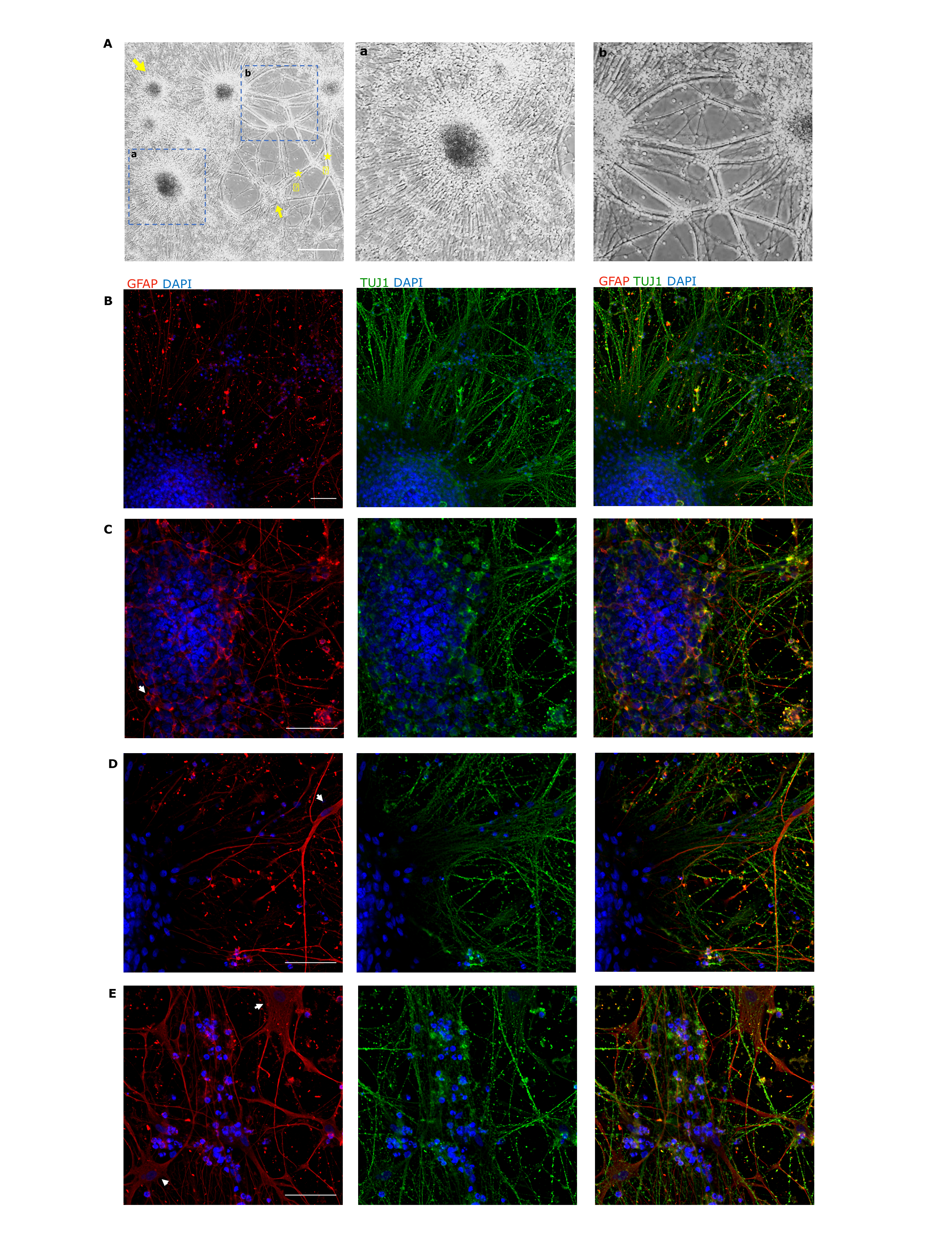


**Figure S2. Representative images of long-term neuronal cultures at 100 days of differentiation, revealing the presence of astrocytes**. A) Phase contrast image. Arrows indicate 3D neuronal aggregates of different sizes and stars show “bundles” of neuronal projections. Scale bar: 500 µm. Magnifications of representative neuronal aggregates (a) and neuronal bundles (b) are shown. (B-E) Representative images of immunostaining for the astrocyte marker GFAP (red) and neuronal marker TUJ1 (green). Images highlight different structures in the neuronal cultures: (B) neuronal aggregates communicating through neuronal bundles at low magnification; (C) medium size neuronal aggregates; (D) small neuronal aggregates and (E) neuronal bundles. Representative astrocytes, covering the bottom of the plate, are indicated with white arrows. Nuclear DAPI staining is shown in blue. Scale bar: 50 µm

**Figure S3. Neurogenic secretome profiling of hiPSCs-derived neuronal cultures.** (A-B) Representative raw data of the neurogenic secretome profile of HC and SZ conditioned media. Red boxes indicate positive internal control spots; blue boxes show negative internal control spots. (C) Heatmap depicting the levels of neurogenic proteins present in four SZ (#1-4) and three HC (#1-3) cell lines at 75 days of differentiation. Data are shown as average level relative to internal control.

**
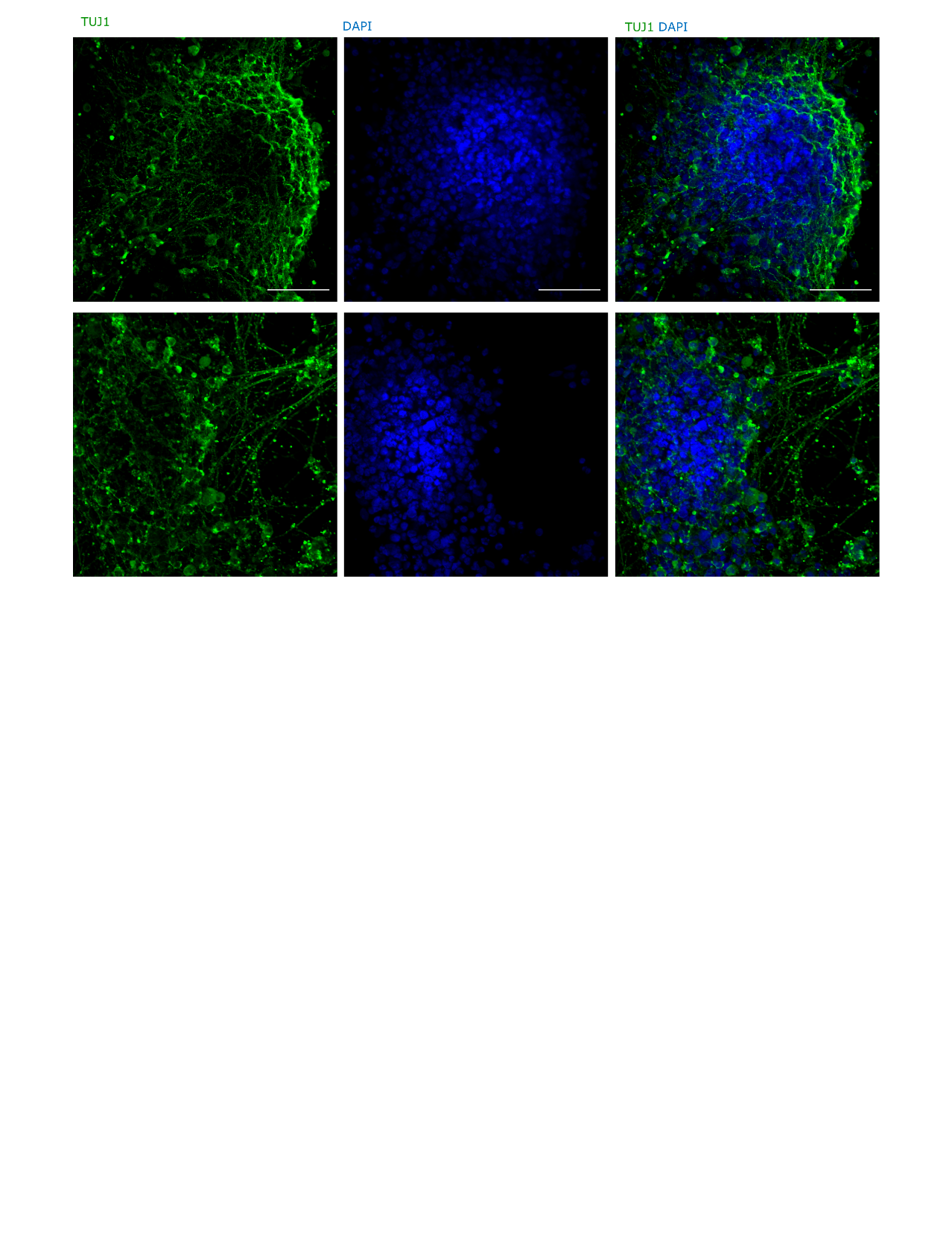
**

**Figure S4. Representative images of long-term neuronal cultures at 100 days of differentiation, revealing the presence of axons in neuronal aggregates.** Immunostaining for the neuronal marker TUJ1 (green) in representative neuronal aggregates show the presence of axons in these structures. Nuclear DAPI staining is shown in blue. Scale bar: 50 µm**.**

**Table S2. Changes in mRNA expression levels along neuronal differentiation.**

| **Gene** | **mean log fold-change (90/30 D) in HC networks** | **fdr adjusted p-value** | **change in HC** | **mean log fold-change (90/30 D) in SZ networks** | **fdr adjusted p-value** | **change in SZ** |
| --- | --- | --- | --- | --- | --- | --- |
| *HOMER1* | -2.07 | 4.59 x10-5 | Decrease | 2.30 | 6.35 x10-4 | Increase |
| *GRIN1* | -7.36 | 1.13 x10-6 | Decrease | 2.28 | 1.06 x10-1 | No change |
| *GPHN* | -6.61 | 2.16 x10-127 | Decrease | -3.37 | 6.89 x10-5 | Decrease |
| *SYP* | 1.26 | 4.49 x10-1 | No change | NA | NA | NA |
| *CDK5R1* | -8.33 | 5.62x10-77 | Decrease | -2.38 | 1.18 x10-2 | Decrease |
| *RELN* | 4.25 | 6.03 x10-2 | No change | -7.55 | 1.44 x10-2 | Decrease |
| *GAD67* | -1.86 | 7.01 x10-2 | No change | 3.79 | 1.72 x10-2 | Increase |
| *GLUT1* | -3.08 | 5.57 x10-2 | No change | 2.63 | 1.33 x10-1 | No change |
| *SEMA3A* | 1.81 | 1.14 x10-1 | No change | 1.10 | 4.26 x10-1 | No change |
| *ATP5* | -1.51 | 9.14 x10-54 | Decrease | 0.14 | 6.8 x10-1 | No change |

Changes in mRNA expression levels of different neurodevelopment-related genes between the 30 and 90 days in culture, for HC and SZ conditions. We estimated the mean log2 fold-change in mRNA expression with an unconditional linear model with a random intercept for cell line identity, to account for the variability within the different cell lines (three SZ (#1, 2, and 3) and two HC (#2 and 3)). With the exception of *SYP* in SZ#1, all measurements were performed in duplicates per cell line. The p-value associated with the mean is obtained from the z-score of the estimated intercept in the linear regression model. If the mean log fold-change 90/30 is significantly larger than zero, we considered an increase in gene expression and vice-versa. For p-value > 0.5, we cannot reject the null hypothesis, suggesting that there was no change in gene expression during the assessed period.

**
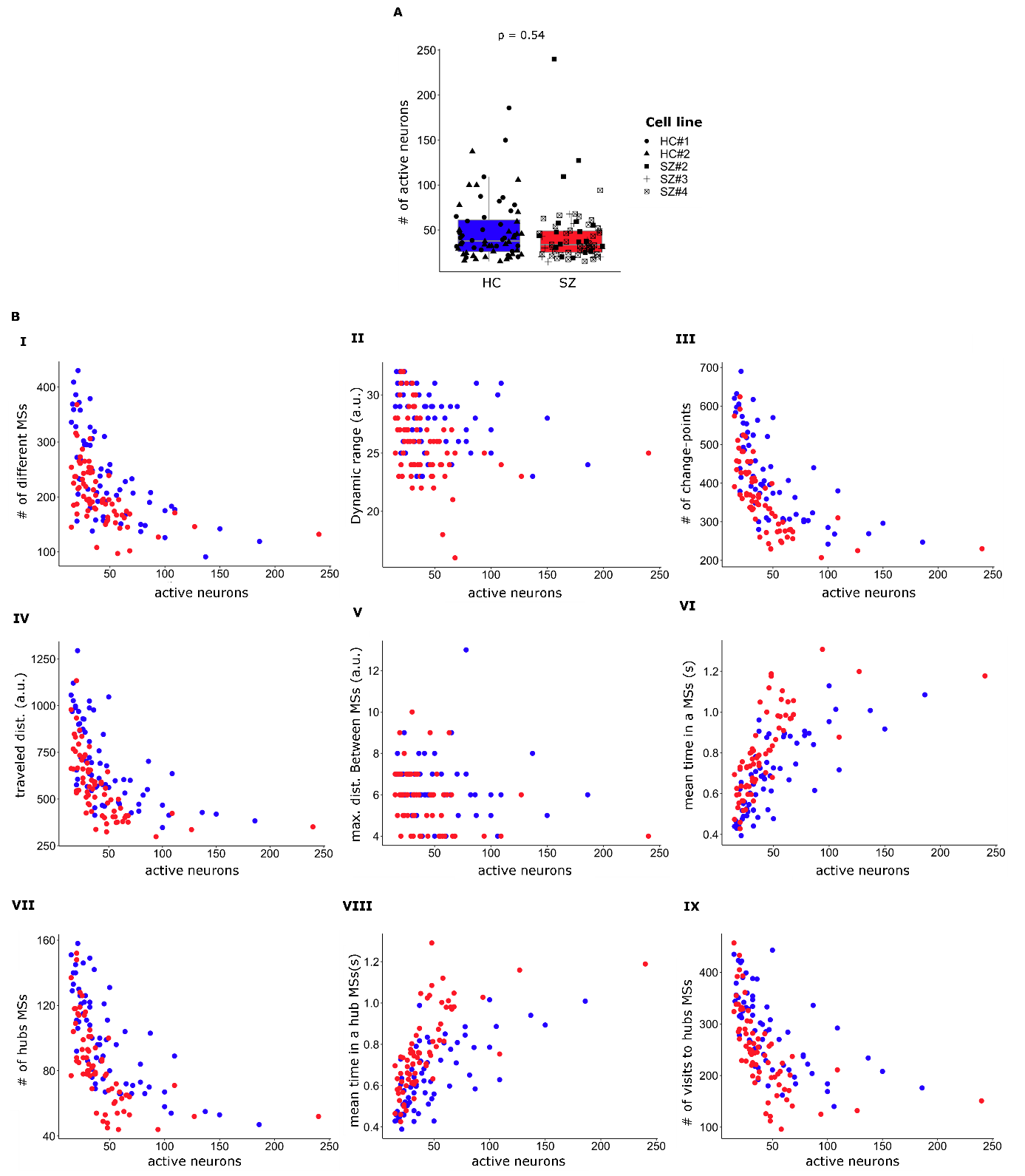
**

**Figure S5. FC-related variables correlate with the number of active neurons**. (A) Boxplots indicating the number of active neurons in the networks, in SZ and HC. Each single data is plotted independently on the boxplot and its symbol indicates the cell line of the particular network. There is no difference in the number of active neurons per network between HC and SZ condition. (B) Scatter plots indicating the relation between the different FC-related variables and the number of active neurons (B.I-B.IX). In most cases, the dependent variable correlated with the number of active neurons in both HC and SZ conditions.


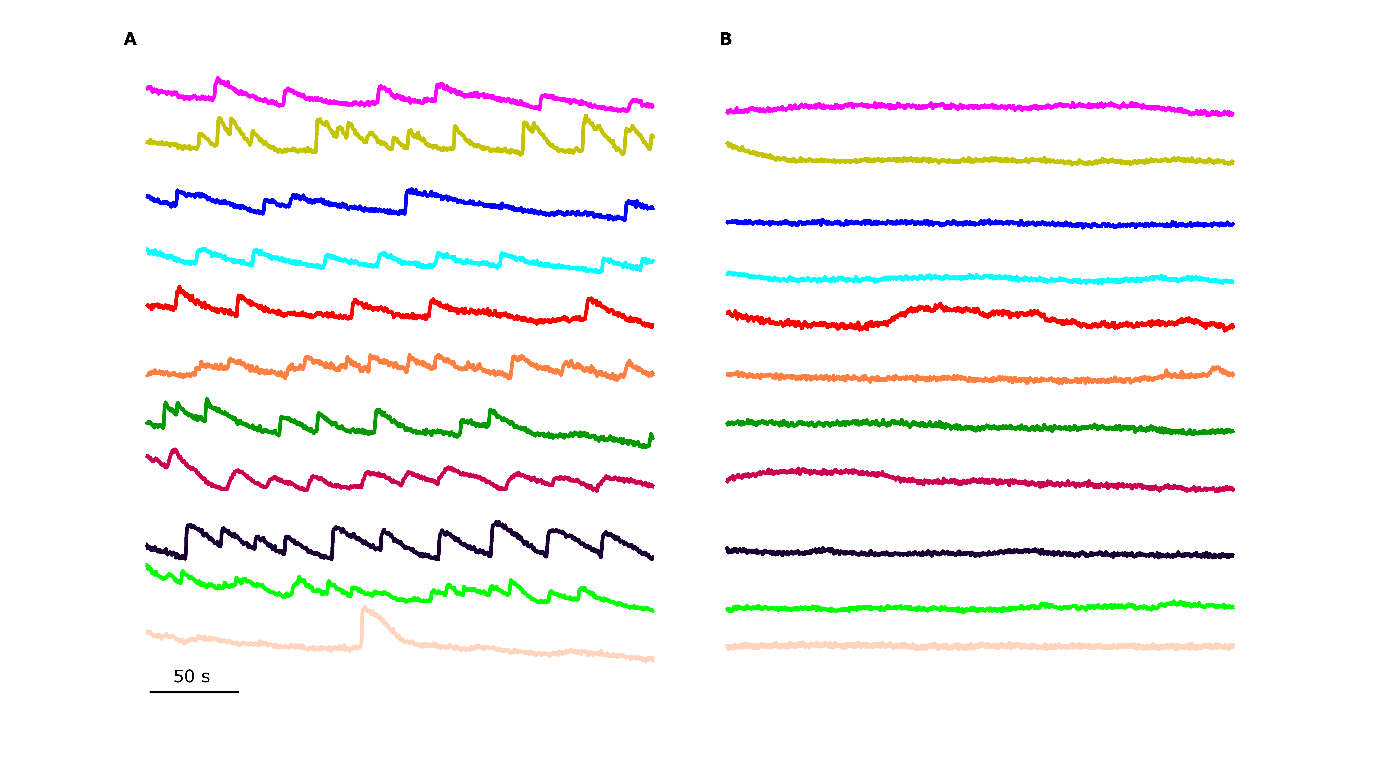


**Figure S6. Ca^2+^ signals rely on voltage-gated Na^+^ channels activation.** Representative Ca^2+^ signals from 11 selected neurons from a neuronal network (A). Events are abolished after adding TTX (0.2 µM) to the bath (B), confirming the AP-dependence of Ca^2+^ transients.

**Table S3. Specifications and output of the mixed effect regression models used to quantify the different FC-related variables (width of the sliding time-window used to identify meta-states = 70 frames, 10.5 s).**

| **Dependent variable** | **intercept (average)** | **estimated SZ effect** | **p-value** | **optimizer** | **included predictors** | **convergence** |
| --- | --- | --- | --- | --- | --- | --- |
| # of active neurons | 49.65 | -6.31 | 5.54 x 10-1 | BFGS | diagnosis, a random intercept per cell line. | yes |
| # of different MSs | 341.95 | -51.47 | 4.43 x 10-3 | CG | diagnosis, # of neurons, # of neurons^2^, a random intercept per cell line. | yes |
| dynamic range (a.u) | 29.69 | -2.46 | 1.08 x 10-3 | BFGS | diagnosis, # of neurons, # of neurons^2^, a random intercept per cell line. | yes |
| # of change-points | 622.06 | -85.38 | 4.85 x 10-9 | BFGS | diagnosis, # of neurons, # of neurons^2^, a random intercept per cell line. | yes |
| mean time in a MS (frames) | 2.12 | 1.06 | 2.96 x 10-6 | BFGS | diagnosis, # of neurons, # of neurons^2^, a random intercept per cell line. | yes |
| max. dist. between MSs (a.u) | 6.37 | -0.69 | 3.17 x 10-2 | BFGS | diagnosis, a random intercept per cell line. | yes |
| traveled dist. (a.u) | 1044.02 | -165.04 | 8.11 x 10-8 | BFGS | diagnosis, # of neurons, # of neurons^2^, a random intercept per cell line. | yes |
| # of hub MSs | 149.57 | -22.31 | 3.1 x 10-6 | CG | diagnosis, # of neurons, # of neurons^2^, a random intercept per cell line. | yes |
| # of visits to hub MSs | 430.11 | -60.17 | 5.28 x 10-3 | CG | diagnosis, # of neurons, # of neurons^2^, a random intercept per cell line. | yes |
| mean time in a hub Ms (frames) | 2.34 | 1.11 | 5.35 x 10-5 | CG | diagnosis, # of neurons, # of neurons^2^, a random intercept per cell line. | yes |

SZ effect and p-value associated with the different variables describing the dynamism of networks functional connectivity (FC), obtained by mixed linear regression modeling (see Methods and Materials). The SZ effect is the coefficient for the diagnosis variable (coded as “1” for SZ and “0” for HC networks). The optimizer and predictors used in each regression model are also shown.

**Table S4. Specifications and output of the mixed effect regression models used to quantify the different FC-related variables (width of the sliding time-window used to identify meta-states = 100 frames, 15.1 s).**

| **Dependent variable** | **intercept**  **(average)** | **estimated SZ effect** | **p-value** | **optimizer** | **included predictors** | **convergence** |
| --- | --- | --- | --- | --- | --- | --- |
| # of active neurons | 49.66 | -6.31 | 5.54 x 10-1 | BFGS | diagnosis, a random intercept per cell line. | yes |
| # of different MSs | 241.8 | -35.8 | 1.7 x 10-3 | CG | diagnosis, # of neurons, # of neurons^2^, a random intercept per cell line. | yes |
| dynamic range (a.u) | 28.78 | -2.81 | 6.76 x 10-5 | BFGS | diagnosis, # of neurons, # of neurons^2^, a random intercept per cell line. | no |
| # of change-points | 423.52 | -65.62 | 8.56 x 10-10 | BFGS | diagnosis, # of neurons, # of neurons^2^, a random intercept per cell line. | yes |
| mean time in a MS (frames) | 2.97 | 1.83 | 4.9 x 10-8 | BFGS | diagnosis, # of neurons, # of neurons^2^, a random intercept per cell line. | yes |
| max. dist. between MSs (a.u) | 5.75 | -0.75 | 8.51 x 10-12 | BFGS | diagnosis, a random intercept per cell line. | yes |
| traveled dist. (a.u) | 671.88 | -120.90 | 3.58 x 10-11 | BFGS | diagnosis, # of neurons, # of neurons^2^, a random intercept per cell line. | no |
| # of hub MSs | 100 | -16.84 | 1.29 x 10-5 | BFGS | diagnosis, # of neurons, # of neurons^2^, a random intercept per cell line. | yes |
| # of visits to hub MSs | 281.84 | -47.71 | 5.71 x 10-9 | CG | diagnosis, # of neurons, # of neurons^2^, a random intercept per cell line. | yes |
| mean time in a hub Ms (frames) | 2.84 | 1.76 | 2.73 x 10-9 | BFGS | diagnosis, # of neurons, # of neurons^2^, a random intercept per cell line. | no |

SZ effect and p-value associated with the different variables describing the dynamism of networks functional connectivity (FC), obtained by mixed linear regression modeling (see Methods and Materials). The SZ effect is the coefficient for the diagnosis variable (coded as “1” for SZ and “0” for HC networks). The optimizer and predictors used in each regression model are also shown.

**Table S5. Specifications and output of the mixed effect regression models used to quantify the different FC-related variables (width of the sliding time-window used to identify meta-states = 200 frames, 30.1 s).**

| **Dependent variable** | **intercept**  **(average)** | **estimated SZ effect** | **p-value** | **optimizer** | **included predictors** | **convergence** |
| --- | --- | --- | --- | --- | --- | --- |
| # of active neurons | 49.66 | -6.31 | 5.54 x 10-1 | BFGS | diagnosis, a random intercept per cell line. | yes |
| # of different MSs | 111.64 | -13.77 | 2.55 x 10-7 | BFGS | diagnosis, # of neurons, # of neurons^2^, a random intercept per cell line. | no |
| dynamic range (a.u) | 25.16 | -1.99 | 5.15 x 10-3 | BFGS | diagnosis, # of neurons, # of neurons^2^, a random intercept per cell line. | yes |
| # of change-points | 176.78 | -23.88 | 1.69 x 10-3 | BFGS | diagnosis, # of neurons, # of neurons^2^, a random intercept per cell line. | yes |
| mean time in a MS (frames) | 7.66 | 3.42 | 6.16 x 10-4 | BFGS | diagnosis, # of neurons, # of neurons^2^, a random intercept per cell line. | yes |
| max. dist. between MSs (a.u) | 4.31 | -0.47 | 7.49 x 10-3 | BFGS | diagnosis, a random intercept per cell line. | no |
| traveled dist. (a.u) | 258.21 | -41.82 | 1.82 x 10-4 | BFGS | diagnosis, # of neurons, # of neurons^2^, a random intercept per cell line. | yes |
| # of hub MSs | 37.54 | -6.03 | 6.35 x 10-2 | BFGS | diagnosis, # of neurons, # of neurons^2^, a random intercept per cell line. | yes |
| # of visits to hub MSs | 103.83 | -15.52 | 1.22 x 10-1 | BFGS | diagnosis, # of neurons, # of neurons^2^, a random intercept per cell line. | yes |
| mean time in a hub Ms (frames) | 6.92 | 3.10 | 6.04 x 10-3 | BFGS | diagnosis, # of neurons, # of neurons^2^, a random intercept per cell line. | yes |

SZ effect and p-value associated with the different variables describing the dynamism of networks functional connectivity (FC), obtained by mixed linear regression modeling (see Methods and Materials). The SZ effect is the coefficient for the diagnosis variable (coded as “1” for SZ and “0” for HC networks). The optimizer and predictors used in each regression model are also shown.

**Annexed Figure.**  Proteome membranes of all original data


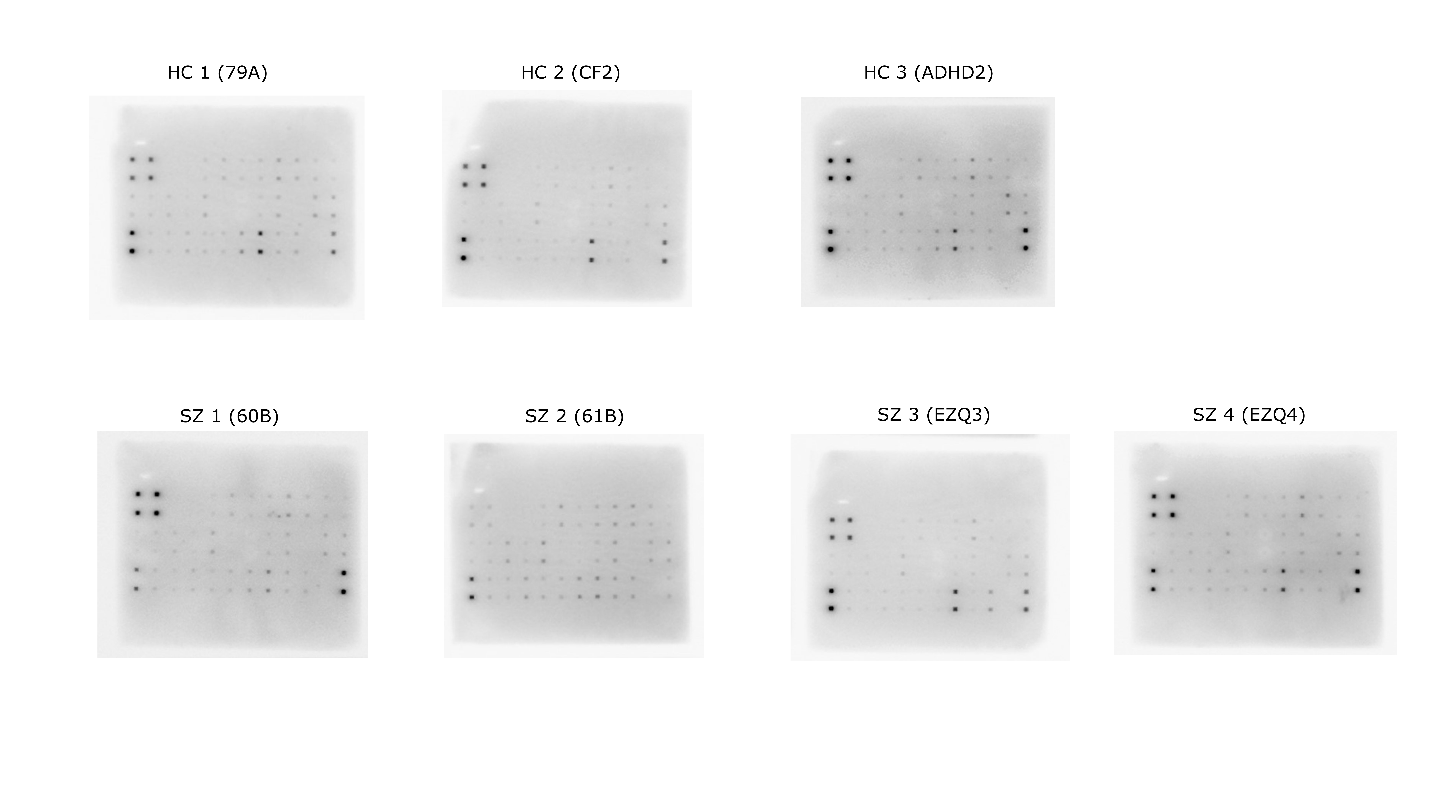


**References**:

1. Brennand, K.J., A. Simone, J. Jou*, et al.*, *Modelling schizophrenia using human induced pluripotent stem cells.* Nature, 2011. **473**(7346): p. 221-225.

2. Casas, B.S., G. Vitória, M.N. do Costa*, et al.*, *hiPSC-derived neural stem cells from patients with schizophrenia induce an impaired angiogenesis.* Transl Psychiatry, 2018. **8**(1): p. 48-48.
